# Bovine coronavirus enters HRT-18 cells via membrane fusion and clathrin-mediated endocytosis in a low pH-, dynamin-, cholesterol-, microtubule-, Rab7-, and Rab11-dependent manner

**DOI:** 10.1128/jvi.01274-25

**Published:** 2025-11-18

**Authors:** Chen Chen, Long Zhao, Nannan Su, Xingyu Peng, Boli Song, Liang Zhang, Kangkang Guo

**Affiliations:** 1 College of Veterinary Medicine, Northwest A&F University 12469, Yangling, Shaanxi, China; 2 College of Animal Science and Technology, Xizang Vocational Technical College 835253 , Lhasa, Xizang, China; University of Kentucky College of Medicine, Lexington, Kentucky, USA

**Keywords:** bovine coronavirus, membrane fusion, clathrin-mediated endocytosis, Rab7, Rab11

## Abstract

Bovine coronavirus (BCoV) infection poses a significant threat to the global cattle industry due to its dual tropism for the respiratory and intestinal systems, causing substantial economic losses. Elucidating the molecular mechanisms of viral entry is critical for developing targeted interventions against BCoV. This study systematically investigates the entry mechanisms of BCoV in HRT-18 cells through different methods. Our data reveal that BCoV entry into HRT-18 cells is dependent on membrane fusion and clathrin-mediated endocytosis (CME). This process is dependent on dynamin, cholesterol, microtubules, cathepsins, and low pH. In contrast, caveolin-mediated endocytosis, micropinocytosis, and TMPRSS2 do not contribute to BCoV entry. Furthermore, we identified Rab7 and Rab11 as key regulators of BCoV endocytosis. Silencing Rab7 and Rab11 significantly inhibited BCoV entry, while silencing Rab5 had no discernible effect. Confocal microscopy confirmed the co-localization of BCoV particles with Rab7 and Rab11, further supporting their role in the viral entry process. Collectively, our findings provide the first evidence that BCoV enters HRT-18 cells via membrane fusion and CME in a low pH-, dynamin-, cholesterol-, microtubule-, cathepsin-, Rab7-, and Rab11-dependent manner. These findings advance our understanding of BCoV pathogenesis and may facilitate the development of novel antiviral strategies against this pathogen.

**IMPORTANCE:**

Emerging and re-emerging coronaviruses are causing severe epidemics in both humans and animals worldwide. Bovine coronavirus (BCoV) is a major pathogen causing severe diarrhea and respiratory disease in cattle, leading to substantial economic losses in the livestock industry. However, the molecular mechanism of BCoV entry into cells remains poorly understood. Here, we reveal that BCoV enters HRT-18 cells via membrane fusion and clathrin-mediated endocytosis, and acidic environment, dynamin, cholesterol, microtubules, cathepsins, Rab7, and Rab11 are also required. This study represents the first report on the mechanism of BCoV cell entry, which advances the understanding of BCoV infection pathogenesis and provides potential targets for the development of novel antiviral drugs.

## INTRODUCTION

Bovine coronavirus (BCoV) is a globally prevalent pathogen that causes severe diarrhea and respiratory disease in cattle, resulting in substantial economic losses to the livestock industry (1). As a member of the family *Coronaviridae*, genus *β-coronavirus*, BCoV is an enveloped, single-stranded positive-sense RNA virus with the longest known RNA genome (~31 kb) (2, 3). This genome contains 13 open reading frames (ORFs) flanked by 5′ and 3′ untranslated regions. The ORF1 encodes a polyprotein, pp1a, which is transferred to pp1ab by ribosomes and then hydrolyzed by proteases into multiple non-structural proteins, which are critical for viral replication and immune evasion (4). BCoV encodes five structural proteins, including spike (S), envelope (E), transmembrane glycoprotein (M), and nucleoprotein (N), and some coronavirus-specific hemagglutinin-esterase (HE) proteins (5). The S protein mediates viral attachment, fusion, and entry (6). The N protein is the only one that binds to the RNA genome and is also involved in viral assembly and budding (7). The M protein plays an important role in BCoV assembly and constitutes the core of the virus together with the N protein (6). The E protein is located inside the M protein and acts together with the M protein in the assembly of viral particles (6, 8). In addition, the E protein also plays a role in the release and pathogenicity of viral particles (8). The HE protein, containing lectin and esterase domains, enhances entry and tropism (9).

Viral infection is initiated by the specific interaction between viral envelope proteins and cellular receptors, followed by viral entry into host cells and the release of viral genomes for replication (9). Thus, viral entry into host cells is a critical determinant of successful infection. Current research indicates that coronaviruses utilize two primary pathways: direct membrane fusion at the plasma membrane or receptor-mediated endocytosis(10). The latter includes clathrin-mediated endocytosis (CME), caveolin-mediated endocytosis (CavME), lipid raft-mediated endocytosis, micropinocytosis, as well as clathrin/caveolin-independent endocytosis . Studies have shown that transmissible gastroenteritis virus enters swine testis (ST) cells via both CME and CavME (11), while porcine epidemic diarrhea virus (PEDV) uses CME, CavME, and lipid raft-dependent endocytosis in African green monkey kidney cells (Vero) and intestinal porcine epithelial cell line-J2 (IPEC-J2) cells (12). Porcine deltacoronavirus (PDCoV) utilizes both macropinocytosis and CME for entry into porcine ileal epithelial cells (IPI-2I), and CavME for ST cells and porcine kidney (PK-15) cells (13, 14). Severe acute respiratory syndrome-related coronavirus (SARS-CoV) employs CME and lipid raft-dependent pathways for host cell entry (15). Collectively, these studies demonstrate that coronaviruses exploit diverse endocytic pathways to invade host cells. However, the precise entry mechanism of BCoV into permissive cells remains poorly characterized.

The endosomal system consists of a series of interconnected membrane chambers responsible for cargo recognition, sorting, trafficking, and degradation, including early endosomes (EEs), late endosomes (LEs), and recycling endosomes (16). Accumulating evidence highlights the critical role of the endosomal system in life cycles, particularly during the entry phase (16, 17). Upon endocytosis, viruses are internalized into endosomes and undergo sequential sorting for intracellular transport. For SARS-CoV-2, the virus binds to the ACE2 receptors at the plasma membrane, triggering endocytosis into Rab5-positive EEs and subsequent maturation into Rab7-positive LEs (18, 19). Similarly, Japanese encephalitis virus exploits Rab5- and Rab11-dependent trafficking from EEs to recycling endosomes, creating a permissive environment for genome release (20). Collectively, these studies highlight the critical role of endosomal trafficking machinery in viral entry.

In this study, chemical inhibitors and RNA interference were employed to investigate which way and molecules were involved in BCoV entry into HRT-18 cells, a permissive cell line for BCoV infection. Our results revealed that BCoV enters HRT-18 cells via membrane fusion and the CME pathway, while CavME and micropinocytosis were not involved. This process was also dependent on an acidic environment, dynamin, cholesterol, microtubules, and cathepsins. Additionally, Rab7 and Rab11 were identified as critical regulators of post-endocytic viral trafficking. These findings provide novel insights into the specific mechanisms of BCoV entry into HRT-18 cells, which may facilitate the development of targeted antiviral strategies.

## RESULTS

### BCoV entry into HRT-18 cells is low pH-dependent

**Fig 1:**
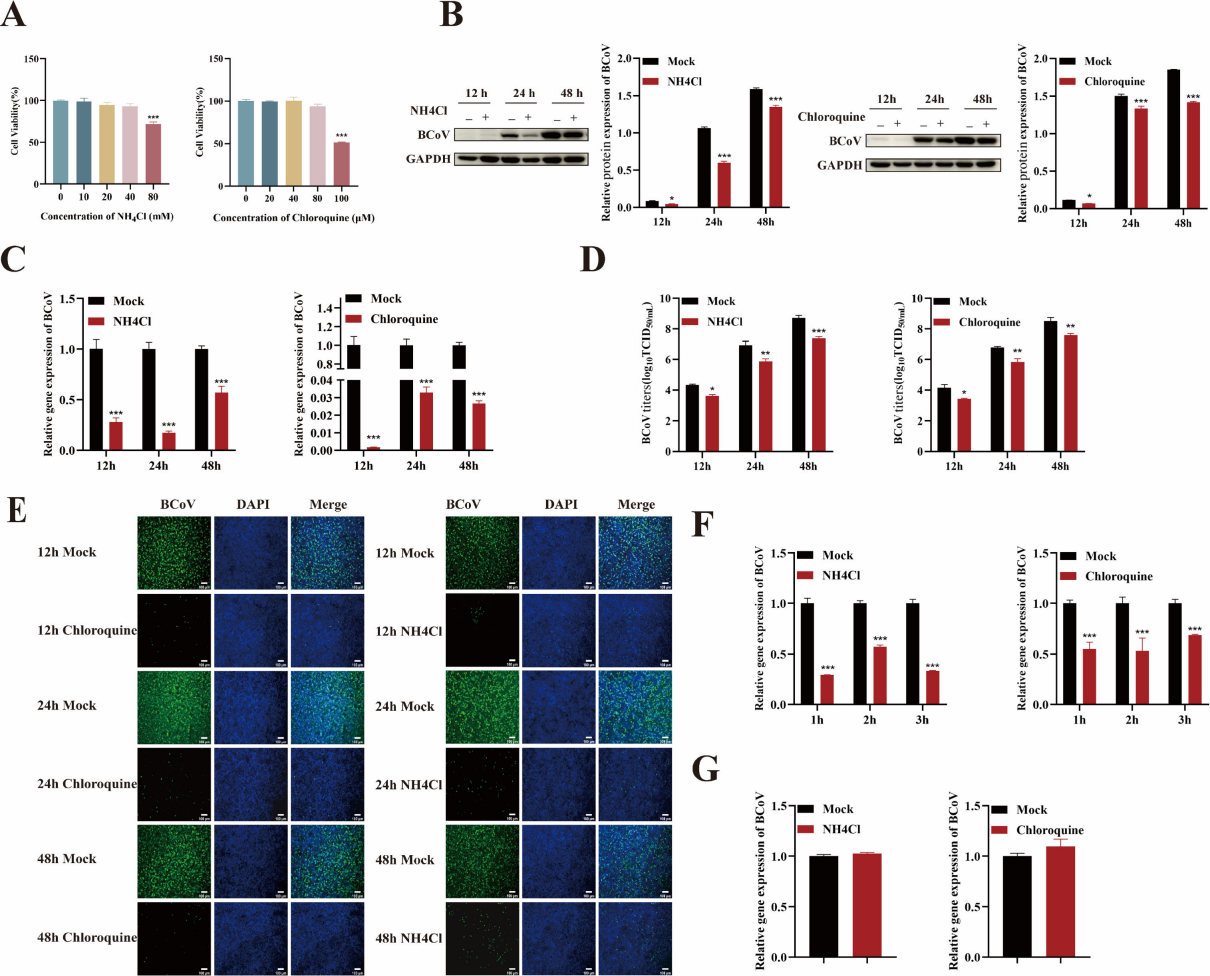
BCoV entry into HRT-18 cells depends on endosomal acidification. (**A**) The maximum safe concentrations of NH_4_Cl and CQ were determined using the CCK-8 assay. (**B**) Western blot analysis was used to evaluate BCoV N protein expression levels, with grayscale analysis performed and presented as a bar graph. (**C**) RT-qPCR was performed to assess the BCoV gene copy numbers. (**D**) TCID_50_ assay was used to measure the BCoV viral titers in the cell supernatant. (**E**) IFA was used to detect the number of BCoV-infected cells. Scale bar = 100 µm. (**F**) RT-qPCR was used to evaluate the effect of NH_4_Cl and CQ on the viral entry. (**G**) RT-qPCR was used to evaluate the effect of NH_4_Cl and CQ on the viral attachment. Data are presented as the mean ± SD of three independent experiments (not significant, *P* > 0.05; *P < 0.05; **P < 0.01; ***P < 0.001).

To investigate the role of an acidic environment in BCoV entry into HRT-18 cells, cells were pretreated with non-toxic concentrations of NH_4_Cl (40 mM) and chloroquine (CQ, 80 µM), both endosomal acidification inhibitors, for 24 h prior to infection with BCoV (MOI = 1) (Fig. 1A). After 12, 24, and 48 h of infection, cells and supernatants were collected for viral proliferation detection. The results showed that treatment with NH_4_Cl or CQ significantly reduced viral RNA levels, N protein expression, and infectious virus titers compared to untreated cells (*P* < 0.05; Fig. 1B through E), indicating that an acidic environment is essential for BCoV infection. To determine whether an acidic environment is required for BCoV entry, cells were pretreated with NH_4_Cl or CQ and infected with BCoV at an MOI of 5 for 1, 2, or 3 h. The RT-qPCR analysis revealed a significant reduction in viral RNA copy numbers in inhibitor-treated cells compared to controls at all time points (*P* < 0.001; Fig. 1F), suggesting that NH_4_Cl and CQ inhibited BCoV entry into HRT-18 cells. To assess the effect of an acidic environment on BCoV attachment, HRT-18 cells were pretreated with CQ and NH_4_Cl for 24 h, followed by incubation with the virus at 4°C for 1 h to allow attachment without internalization. RT-qPCR analysis showed that neither CQ nor NH_4_Cl treatment affected BCoV attachment to the cell surface (*P* > 0.05; Fig. 1G). These results confirmed that an acidic environment is essential for BCoV entry into HRT-18 cells but not for viral attachment.

### BCoV entry into HRT-18 cells depends on dynamin

**Fig 2:**
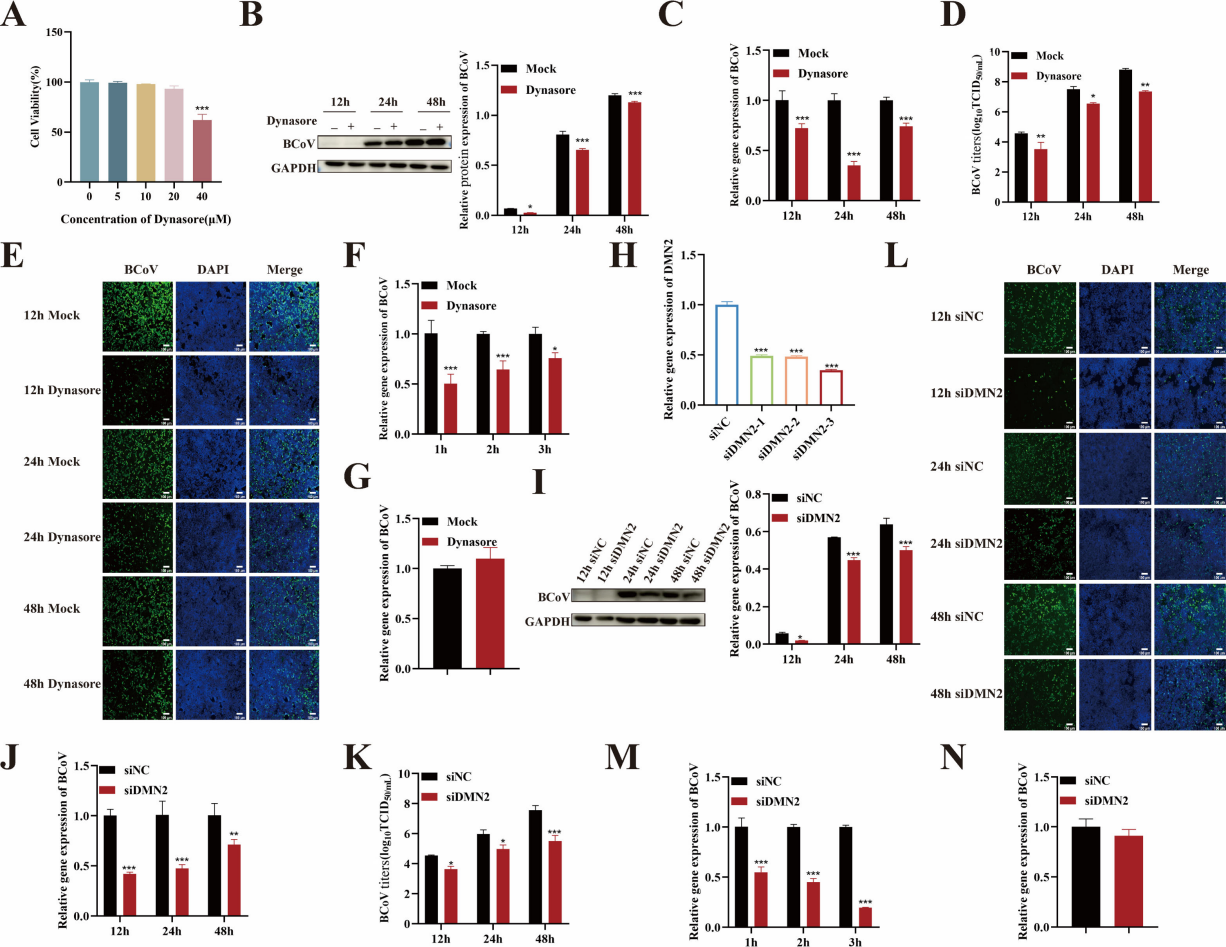
BCoV entry into HRT-18 cells depends on dynamin. (**A**) The maximum safe concentrations of dynasore were determined using the CCK-8 assay. (**B**) Western blot analysis was used to evaluate the BCoV N protein expression levels, with grayscale analysis performed and presented as a bar graph. (**C**) RT-qPCR was performed to assess the BCoV gene copy numbers. (**D**) TCID_50_ assay was used to measure the BCoV viral titers in the cell supernatant. (**E**) IFA was used to detect the number of BCoV-infected cells. Scale bar = 100 µm. (**F**) RT-qPCR was used to evaluate the effect of dynasore on the viral entry. (**G**) RT-qPCR was used to evaluate the effect of dynasore on the viral attachment. (**H**) The siRNA silencing efficiency of dynamin was screened; the effects of dynamin-silenced cells on BCoV infection were assessed by (**I**) Western blot, (**J**) RT-qPCR, (**K**) TCID_50_, and (**L**) IFA. (**M**) RT-qPCR was used to evaluate the effect of dynamin-silenced cells on the viral entry; (**N**) RT-qPCR was used to evaluate the effects of dynamin-targeting siRNA on BCoV attachment. Data are presented as the mean ± SD of three independent experiments (not significant, *P* > 0.05; *P < 0.05; **P < 0.01; ***P < 0.001).

Dynamin, a GTPase critical for endocytosis (including clathrin-mediated and caveolin-mediated pathways), has been implicated in viral entry (21, 22). To investigate the potential role of dynamin in BCoV infection and entry, dynamin inhibitor dynasore was used. Cytotoxicity assays revealed that 20 µM dynasore was non-toxic to HRT-18 cells (Fig. 2A). Subsequently, cells were pretreated with 20 µM dynasore for 24 h and infected with BCoV (MOI = 1). Viral replication was assessed at 12, 24, and 48 h post-infection via viral RNA quantification, N protein analysis, and infectious virus titration. We observed that BCoV infection was markedly inhibited in dynasore-treated cells (*P* < 0.05; Fig. 2B through E). To determine whether dynamin affects BCoV entry, cells were pretreated with dynasore and infected with BCoV at an MOI of 5 for 1, 2, or 3 h. RT-qPCR analysis revealed a significant reduction in viral RNA copy numbers in dynasore-treated cells at all time points (*P* < 0.05; Fig. 2F), indicating that dynamin is required for the viral entry. Dynasore treatment had no effect on BCoV attachment to the cell surface, indicating that dynamin is not involved in the attachment process (*P* > 0.05; Fig. 2G). To further evaluate the role of dynamin in BCoV endocytosis, we used siRNA to silence dynamin expression. The silencing efficiency was assessed by RT-qPCR, and the siRNA with the highest silencing efficiency was selected for subsequent experiments (Fig. 2H). HRT-18 cells were transfected with dynamin-targeting siRNA for 48 h, followed by BCoV infection for 12, 24, and 48 h. The results showed that viral RNA copy numbers, N protein expression, and infectious virus titers were significantly reduced (*P* < 0.05; Fig. 2I through L), indicating that silencing dynamin markedly inhibited BCoV infection. The effects of dynamin silencing on BCoV attachment and entry were subsequently evaluated. RT-qPCR analysis showed a significant reduction in viral RNA copy numbers during the entry phase (*P* < 0.01; Fig. 2M), whereas no significant changes were observed during the attachment phase (*P* > 0.05; Fig. 2N). These findings indicate that dynamin is required for BCoV entry but does not affect the initial attachment to HRT-18 cells.

### BCoV entry into HRT-18 cells via membrane fusion

**Fig 3:**
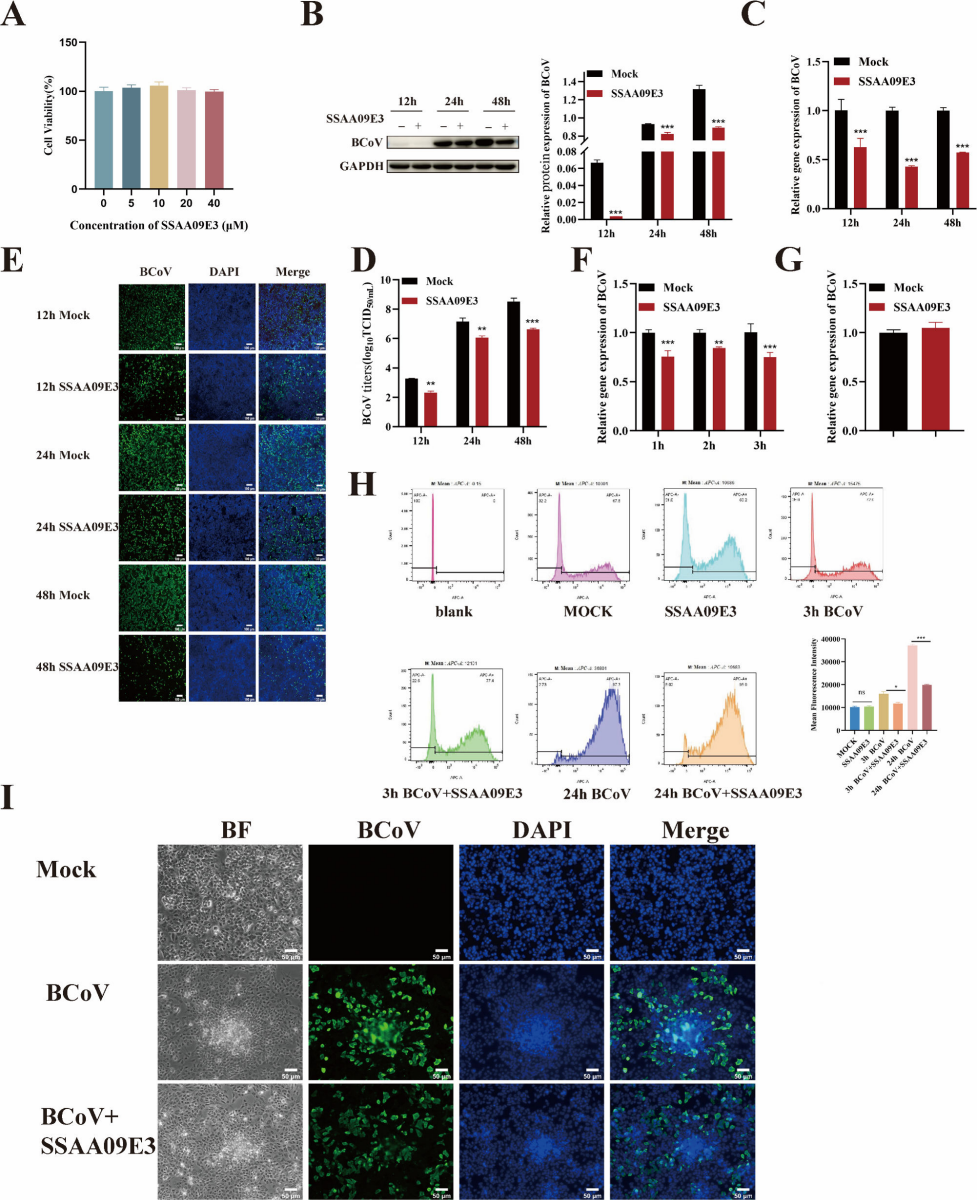
BCoV entry into HRT-18 cells with membrane fusion dependence. (**A**) The maximum safe concentration of SSAA09E3 was determined by CCK-8 assay. (**B**) Western blot analysis was used to evaluate the BCoV N protein expression levels, with grayscale analysis performed and presented as a bar graph. (**C**) RT-qPCR was performed to assess the BCoV gene copy numbers. (**D**) TCID_50_ assay was used to measure the BCoV viral titers in the cell supernatant. (**E**) IFA was used to detect the number of BCoV-infected cells. Scale bar = 100 µm. (**F**) RT-qPCR was used to evaluate the effect of SSAA09E3 on the viral entry. (**G**) RT-qPCR was used to evaluate the effect of SSAA09E3 on the viral attachment. (**H**) Histogram and MFI analysis of DiD fluorescence in the APC channel detected by flow cytometry. (**I**) SSAA09E3 inhibits BCoV-induced syncytium formation in HRT-18 cells. Scale bar = 50 µm. Data are presented as the mean ± SD of three independent experiments (not significant, *P* > 0.05; *P < 0.05; **P < 0.01; ***P < 0.001).

Membrane fusion is a crucial step in the entry of enveloped viruses into host cells, particularly for coronaviruses (10). To evaluate the role of membrane fusion in BCoV entry, we tested the novel fusion inhibitor SSAA09E3. The CCK-8 assays confirmed that 40 µM SSAA09E3 exhibited no cytotoxicity in HRT-18 cells (Fig. 3A). Cells were pretreated with 40 µM SSAA09E3 for 24 h prior to infection with BCoV (MOI = 1). Viral propagation was analyzed at 12, 24, and 48 h post-infection by quantifying viral RNA copy numbers, N protein expression, infection rates, and progeny virus titers. Figure 3B through E demonstrated that SSAA09E3 treatment significantly reduced BCoV gene copy numbers, N protein expression levels, viral infection rate, and progeny virus titer compared to controls (*P* < 0.01), indicating effective inhibition of BCoV propagation. To assess whether SSAA09E3 targets viral entry, cells were pretreated with the inhibitor and infected with BCoV (MOI = 5) for 1, 2, or 3 h. RT-qPCR analysis revealed a significant reduction in viral RNA copy numbers in SSAA09E3-treated cells at all time points (*P* < 0.01; Fig. 3F), confirming that SSAA09E3 inhibits BCoV entry. We also showed that inhibition of membrane fusion did not affect BCoV attachment to the cell surface (*P* > 0.05; Fig. 3G). Subsequently, flow cytometry analysis demonstrated that SSAA09E3 significantly inhibited BCoV entry by suppressing syncytium formation (Fig. 3H). Further microscopic visualization revealed that BCoV infection induces syncytium formation in HRT-18 cells and that SSAA09E3 treatment significantly inhibits this process (Fig. 3I). Collectively, these findings establish membrane fusion as an essential mechanism for BCoV entry into HRT-18 cells, while having no effect on viral attachment.

### BCoV entry depends on clathrin-mediated endocytosis

**Fig 4:**
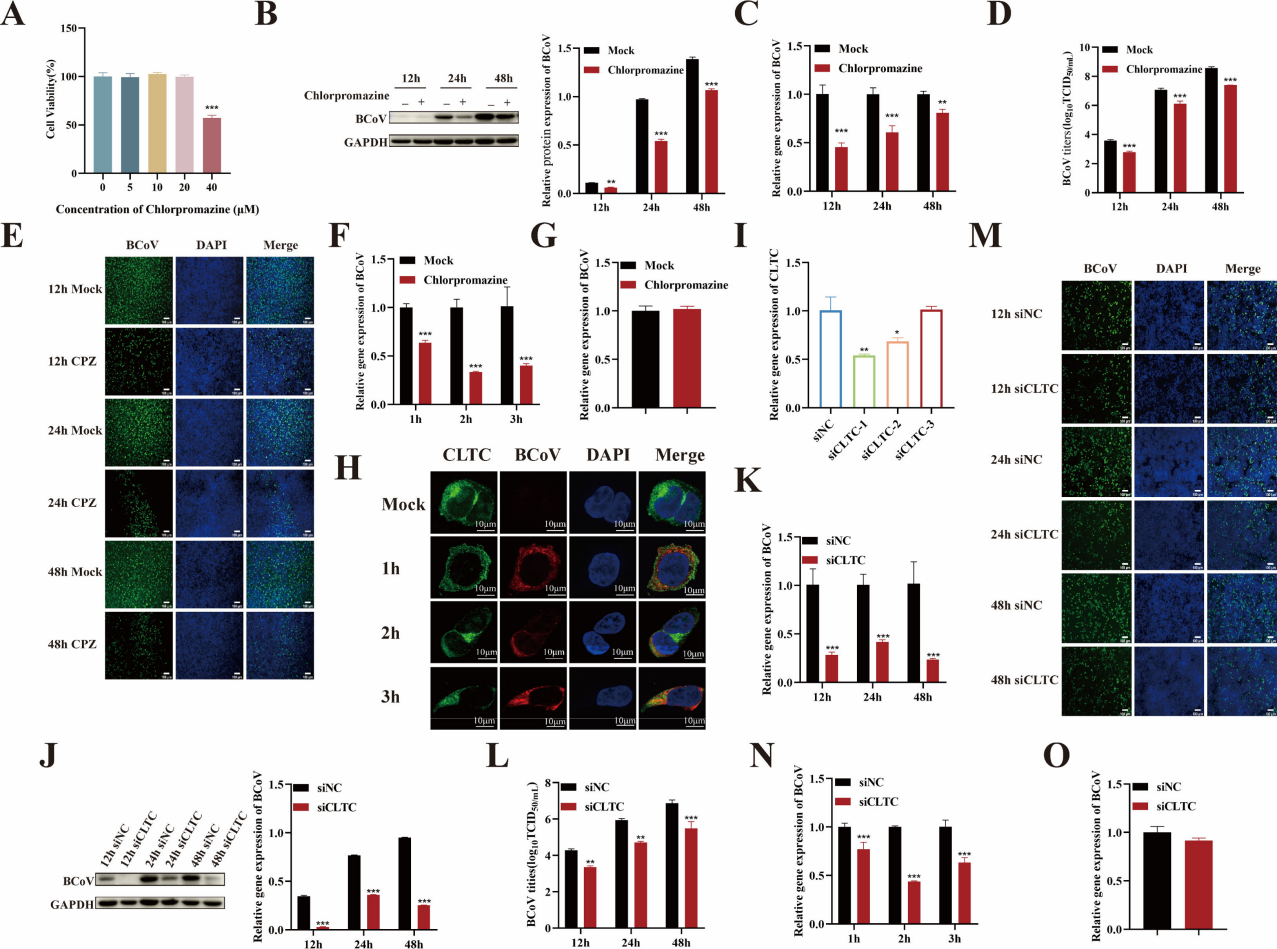
BCoV enters HRT-18 cells through a clathrin-mediated endocytosis pathway. (**A**) Determination of the maximum safe concentration of CPZ using the CCK-8 assay. (**B**) Western blot analysis was used to evaluate the BCoV N protein expression levels, with grayscale analysis performed and presented as a bar graph. (**C**) RT-qPCR was performed to assess the BCoV gene copy numbers. (**D**) TCID_50_ assay was used to measure the BCoV viral titers in the cell supernatant. (**E**) IFA was used to detect the number of BCoV-infected cells. Scale bar = 100 µm. (**F**) RT-qPCR was used to evaluate the effect of CPZ on the viral entry. (**G**) Confocal microscopy images showing co-localization of BCoV and clathrin during the entry phase. Scale bar = 10 µm. (**H**) RT-qPCR was used to evaluate the effect of CPZ on the viral attachment. (**I**) The siRNA silencing efficiency of CLTC was screened; the effects of clathrin-silenced cells on BCoV infection were assessed by (**J**) Western blot, (**K**) RT-qPCR, (**L**) TCID_50_, and (**M**) IFA. (**N**) RT-qPCR was used to evaluate the effect of CLTC-silenced cells on the viral entry; (**O**) RT-qPCR was used to evaluate the effects of CLTC-targeting siRNA on BCoV attachment. Data are presented as the mean ± SD of three independent experiments (not significant, *P* > 0.05; *P < 0.05; **P < 0.01; ***P < 0.001).

Clathrin-mediated endocytosis (CME), the classical endocytic pathway, is exploited by numerous viruses for cellular entry (23). To determine whether BCoV entry relies on CME, we used chlorpromazine (CPZ, 20 µM) (Fig. 4A), a specific inhibitor of CME, to pretreated HRT-18 cells for 24 h. Subsequently, cells were infected with BCoV (MOI = 1) for 12, 24, and 48 h. Viral proliferation was assessed by quantifying viral RNA copy numbers and N protein expression in cell lysates, and infectious virus titers in supernatants. The results revealed that CPZ significantly inhibited viral infection (*P* < 0.01) (Fig. 4B through E). To evaluate CPZ’s effect on viral entry, pretreated cells were infected with BCoV (MOI of 5) for 1, 2, and 3 h. The results revealed a significant reduction in viral RNA copy numbers in CPZ-treated cells at all time points (*P* < 0.001; Fig. 4F), confirming inhibition of BCoV entry. Confocal microscopy further demonstrated co-localization of BCoV with clathrin during the entry phase (Fig. 4G). Subsequently, the effect of CPZ on BCoV attachment was evaluated. As shown in Fig. 4H, no significant difference in viral RNA copy numbers was observed between the control and CPZ-treated groups (*P* > 0.05), indicating that CPZ had no effect on BCoV attachment. To further confirm the role of CME in BCoV entry steps, clathrin heavy chain (CLTC) expression was silenced by siRNA. Silencing efficiency was evaluated by RT-qPCR, and the siRNA with the highest efficiency was selected for subsequent experiments (Fig. 4I). HRT-18 cells were transfected with siCLTC for 48 h, followed by BCoV infection. Samples were collected at 12, 24, and 48 h post-infection. Viral RNA copy numbers were quantified by RT-qPCR, N protein expression was analyzed by Western blotting, and viral infection rate and titers were determined by IFA andtissue culture infective dose 50 ( TCID_50_) assay (*P* < 0.01; Fig. 4J through M). The results showed that silencing of CLTC significantly inhibited BCoV infection. Subsequently, the impact of CLTC silencing on BCoV attachment and entry was evaluated. RT-qPCR analysis revealed a significant reduction in viral RNA copy numbers during the entry phase (*P* < 0.001; Fig. 4N), but not in the attachment phase (*P* > 0.05; Fig. 4O). Collectively, these results indicate that BCoV entry into HRT-18 cells requires CME.

### BCoV entry into HRT-18 cells is caveolin-mediated endocytosis and macropinocytosis independent

**Fig 5:**
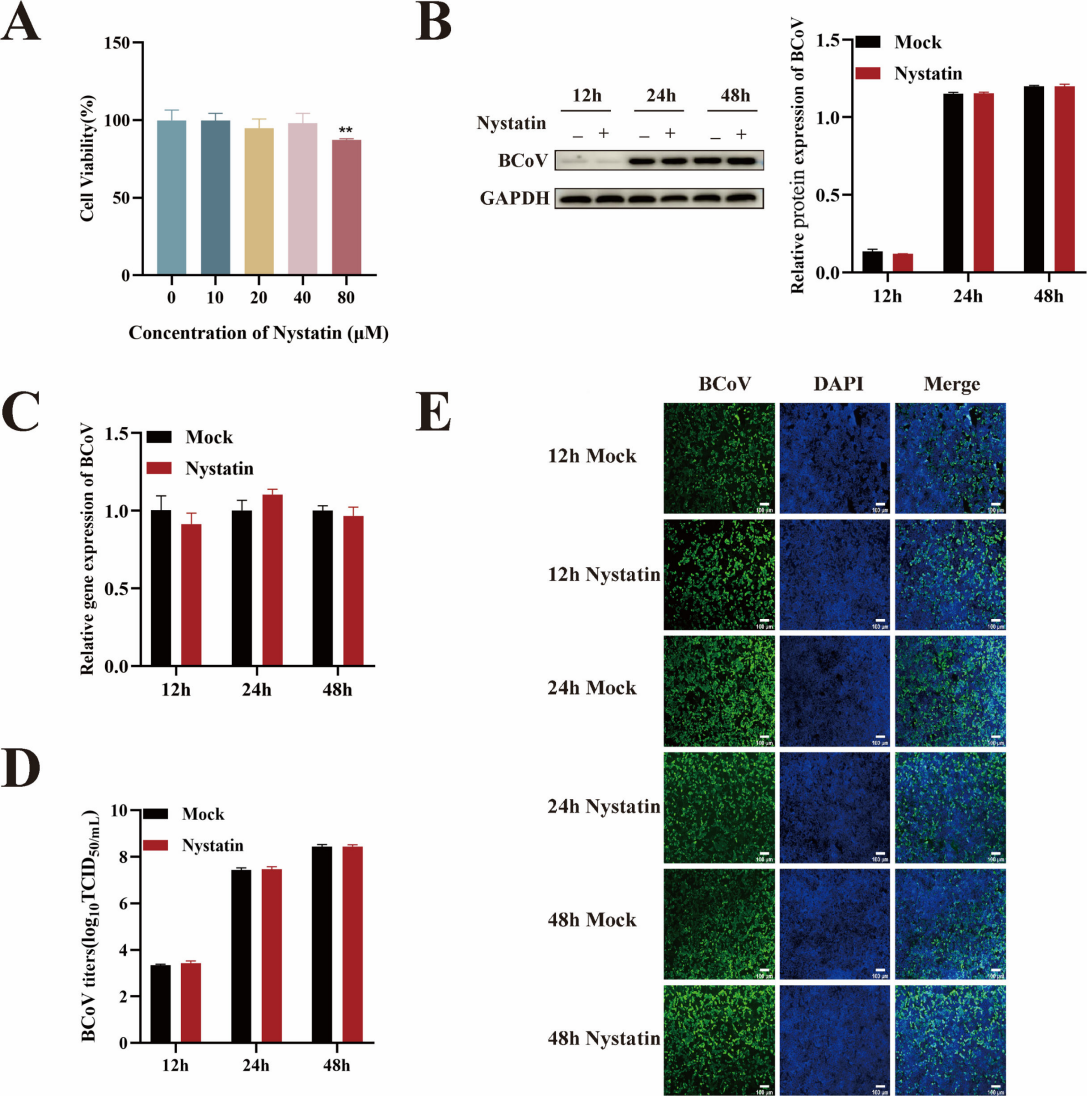
The entry of BCoV into HRT-18 cells was not mediated by the CavME pathway. (**A**) The maximum safe concentrations of nystatin were determined using the CCK-8 assay. (**B**) Western blot analysis was used to evaluate the BCoV N protein expression levels, with grayscale analysis performed and presented as a bar graph. (**C**) RT-qPCR was performed to assess the BCoV gene copy numbers. (**D**) TCID_50_ assay was used to measure the BCoV viral titers in the cell supernatant. (**E**) IFA was used to detect the number of BCoV-infected cells. Scale bar = 100 µm. Data are presented as the mean ± SD of three independent experiments (not significant, *P* > 0.05).

Caveolin-mediated endocytosis (CavME) is another important endocytic pathway, different from CME (24), that was investigated for its potential role in BCoV infection. To determine whether BCoV utilizes CavME, we employed the CavME inhibitor nystatin. A CCK-8 assay identified 80 µM nystatin as the maximum non-cytotoxic concentration (Fig. 5A). Next, HRT-18 cells were pretreated with nystatin (80 µM) for 24 h and infected with BCoV (MOI = 1) to assess the role of CavME in viral infection. Cell samples and supernatants were collected at 12, 24, and 48 h post-infection. Analyses were conducted using RT-qPCR, Western blotting, IFA, and TCID_50_. The results suggest that nystatin had no significant impact on BCoV proliferation (*P* > 0.05; Fig. 5B through E), suggesting that BCoV entry into HRT-18 cells does not rely on CavME.

**Fig 6:**
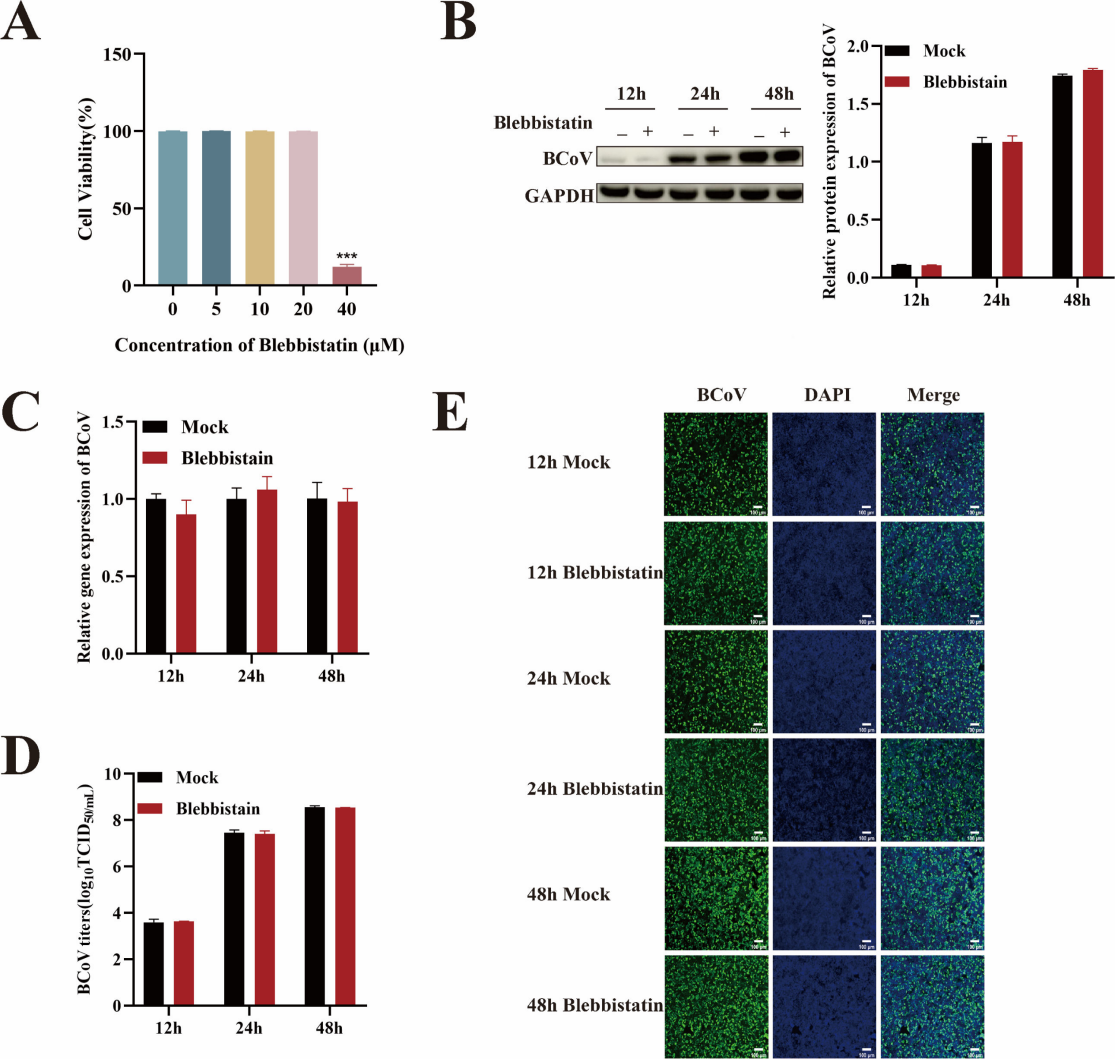
BCoV invasion of HRT-18 cells does not depend on the macropinocytosis pathway. (**A**) The maximum safe concentrations of blebbistatin were determined using the CCK-8 assay. (**B**) Western blot analysis was used to evaluate the BCoV N protein expression levels, with grayscale analysis performed and presented as a bar graph. (**C**) RT-qPCR was performed to assess the BCoV gene copy numbers. (**D**) TCID_50_ assay was used to measure the BCoV viral titers in the cell supernatant. (**E**) IFA was used to detect the number of BCoV-infected cells. Scale bar = 100 µm. Data are presented as the mean ± SD of three independent experiments (not significant, *P* > 0.05).

Macropinocytosis, a non-specific endocytic pathway, has been implicated in viral entry for certain pathogens. To investigate whether micropinocytosis contributes to BCoV entry, the macropinocytosis inhibitor blebbistatin was utilized (25). Cells were pretreated with maximum safe concentration of blebbistatin (20 µM) for 24 h and subsequently infected with BCoV (MOI = 1). The BCoV gene copy numbers, protein expression levels, viral infection numbers, and progeny viral titers were measured by RT-qPCR, Western blot, IFA, and TCID_50_ after infection for 12, 24, and 48 h. The results demonstrated no significant impact of blebbistatin treatment on BCoV propagation (*P* > 0.05; Fig. 6A through E). These findings collectively confirm that blebbistatin did not affect BCoV infection and indicate that BCoV entry into HRT-18 cells is independent of micropinocytosis.

### The effect of cholesterol on BCoV entry into HRT-18 cells

**Fig 7:**
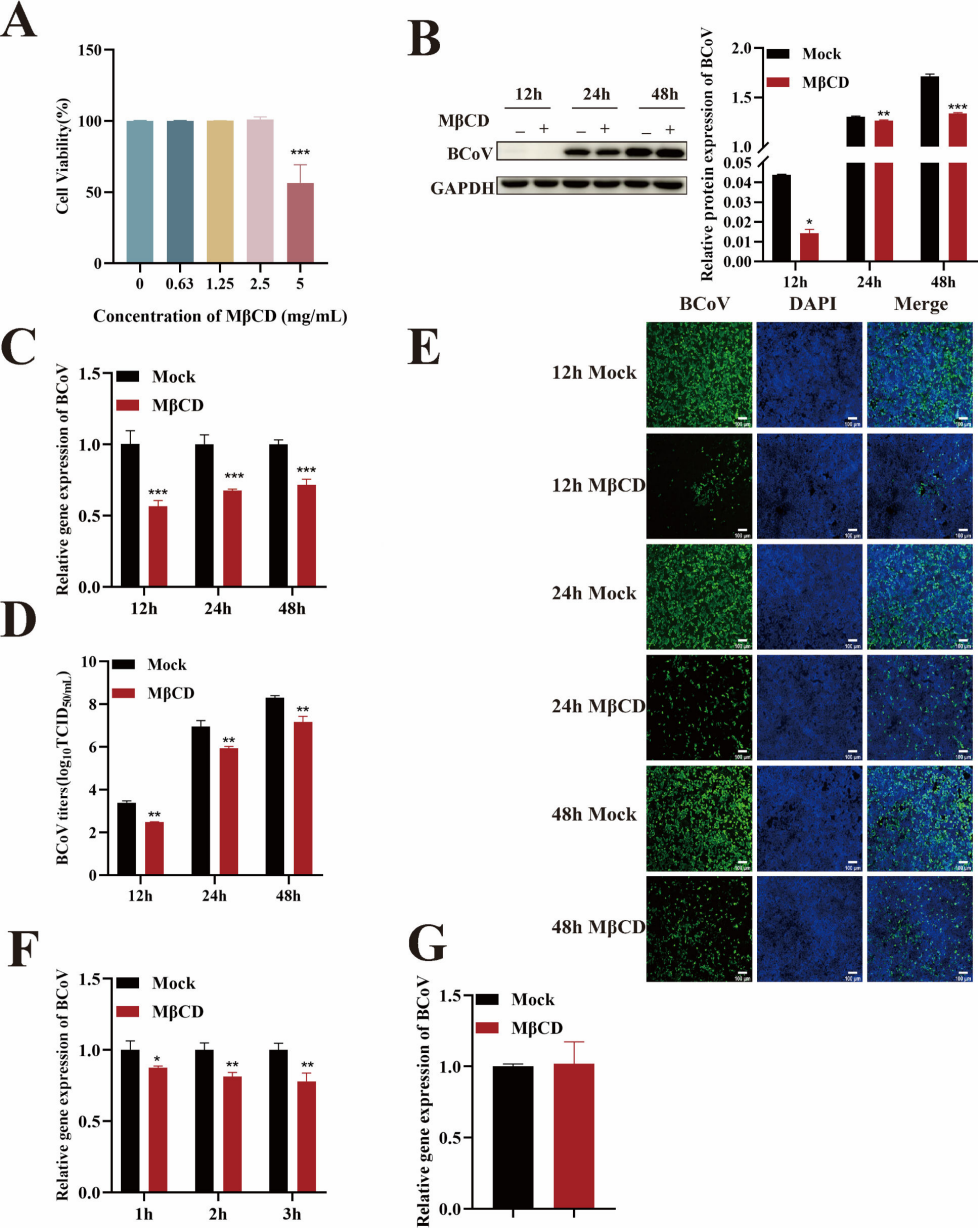
BCoV requires cholesterol to enter HRT-18 cells. (**A**) The maximum safe concentrations of MβCD were determined using the CCK-8 assay. (**B**) Western blot analysis was used to evaluate the BCoV N protein expression levels, with grayscale analysis performed and presented as a bar graph. (**C**) RT-qPCR was performed to assess the BCoV gene copy numbers. (**D**) TCID_50_ assay was used to measure the BCoV viral titers in the cell supernatant. (**E**) IFA was used to detect the number of BCoV-infected cells. Scale bar = 100 µm. (**F**) RT-qPCR was used to evaluate the effect of MβCD on the viral entry. (**G**) RT-qPCR was used to evaluate the effect of MβCD on the viral attachment. Data are presented as the mean ± SD of three independent experiments (not significant, *P* > 0.05; *P < 0.05; **P < 0.01; ***P < 0.001).

Cholesterol plays an essential role in the viral life cycle, and many coronaviruses require cholesterol in the viral envelope or host cell membrane for cellular entry (26). Here, methyl-β-cyclodextrin (MβCD), a cholesterol depletion agent, was used to analyze the role of cholesterol in BCoV infection and entry. The CCK-8 assays confirmed that 2.5 mg/mL MβCD exhibited no cytotoxicity in HRT-18 cells (Fig. 7A). Cells were pretreated with MβCD (2.5 mg/mL) for 24 h and infected with BCoV (MOI = 1). Viral infection was evaluated at 12, 24, and 48 h post-infection by quantifying viral RNA copy numbers, N protein expression, infection rates, and progeny virus titers (*P* < 0.05; Fig. 7B through E), indicating that cholesterol is required for BCoV infection. To assess the role of cholesterol in BCoV entry and attachment, cells were pretreated with MβCD (2.5 mg/mL) for 24 h and then infected with BCoV. RT-qPCR analysis revealed that cholesterol depletion significantly reduced BCoV entry (*P* < 0.05; Fig. 7F) but had no effect on viral attachment (*P* > 0.05; Fig. 7G), indicating that BCoV entry into HRT-18 cells is cholesterol-dependent.

### Microtubule is required for BCoV entry into HRT-18 cells

**Fig 8:**
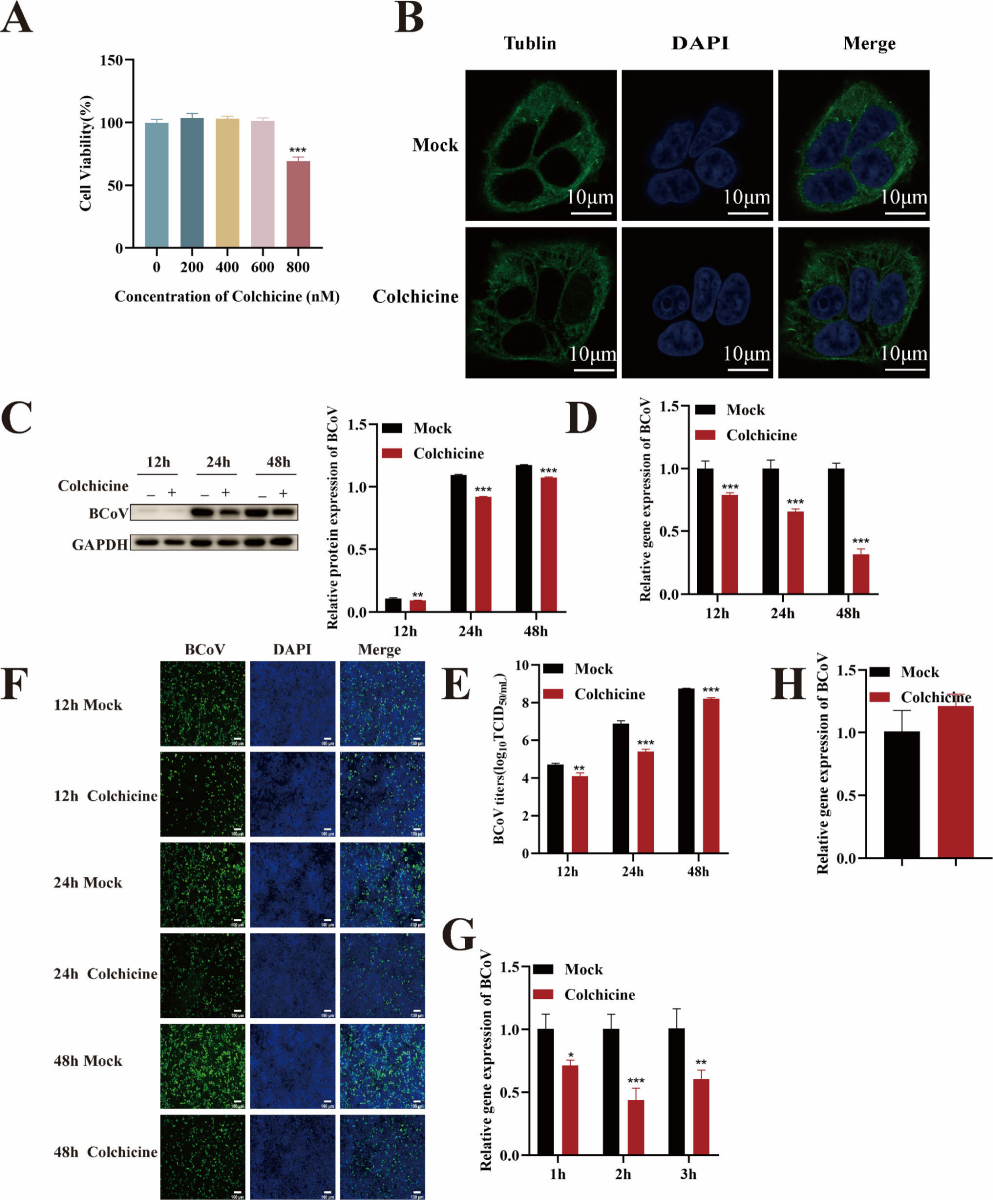
BCoV entry into HRT-18 cells depends on the stability of the microtubule system. (**A**) The maximum safe concentrations of colchicine were determined using the CCK-8 assay. (**B**) Microtubule depolymerization was observed by confocal microscopy. Scale bar = 10 µm. (**C**) Western blot analysis was used to evaluate the BCoV N protein expression levels, with grayscale analysis performed and presented as a bar graph. (**D**) RT-qPCR was performed to assess the BCoV gene copy numbers. (**E**) TCID_50_ assay was used to measure the BCoV viral titers in the cell supernatant. (**F**) IFA was used to detect the number of BCoV-infected cells. Scale bar = 100 µm. (**G**) RT-qPCR was used to evaluate the effect of colchicine on the viral entry. (**H**) RT-qPCR was used to evaluate the effect of colchicine on the viral attachment. Data are presented as the mean ± SD of three independent experiments (not significant, *P* > 0.05; *P < 0.05; **P < 0.01; ***P < 0.001).

Upon internalization, viruses traffic through endosomes along microtubule tracks. To investigate whether BCoV hijacks microtubules for intracellular transport, we disrupted microtubule polymerization using colchicine, a potent microtubule depolymerizing agent. Firstly, the maximum safe concentration of colchicine was determined to be 600 nM by CCK-8 assay (Fig. 8A). Confocal microscopy revealed that 600 nM colchicine induced microtubule depolymerization, disrupting the compact microtubule network into fragmented structures (Fig. 8B). HRT-18 cells were pretreated with 600 nM colchicine for 24 h prior to infection with BCoV (MOI = 1). Viral replication was evaluated at 12, 24, and 48 h post-infection via RT-qPCR (viral RNA copy numbers), Western blotting (N protein expression), IFA (infection rates), and TCID_50_ (progeny titers). Colchicine treatment significantly reduced BCoV propagation (*P* < 0.01; Fig. 8C through F). RT-qPCR analysis revealed that colchicine treatment significantly reduced BCoV entry (*P* < 0.05; Fig. 8G) but had no effect on viral attachment (*P* > 0.05; Fig. 8H), indicating that microtubule integrity is essential for BCoV entry into HRT-18 cells but dispensable for attachment.

### Cathepsins are essential for BCoV entry into HRT-18 cells

**Fig 9:**
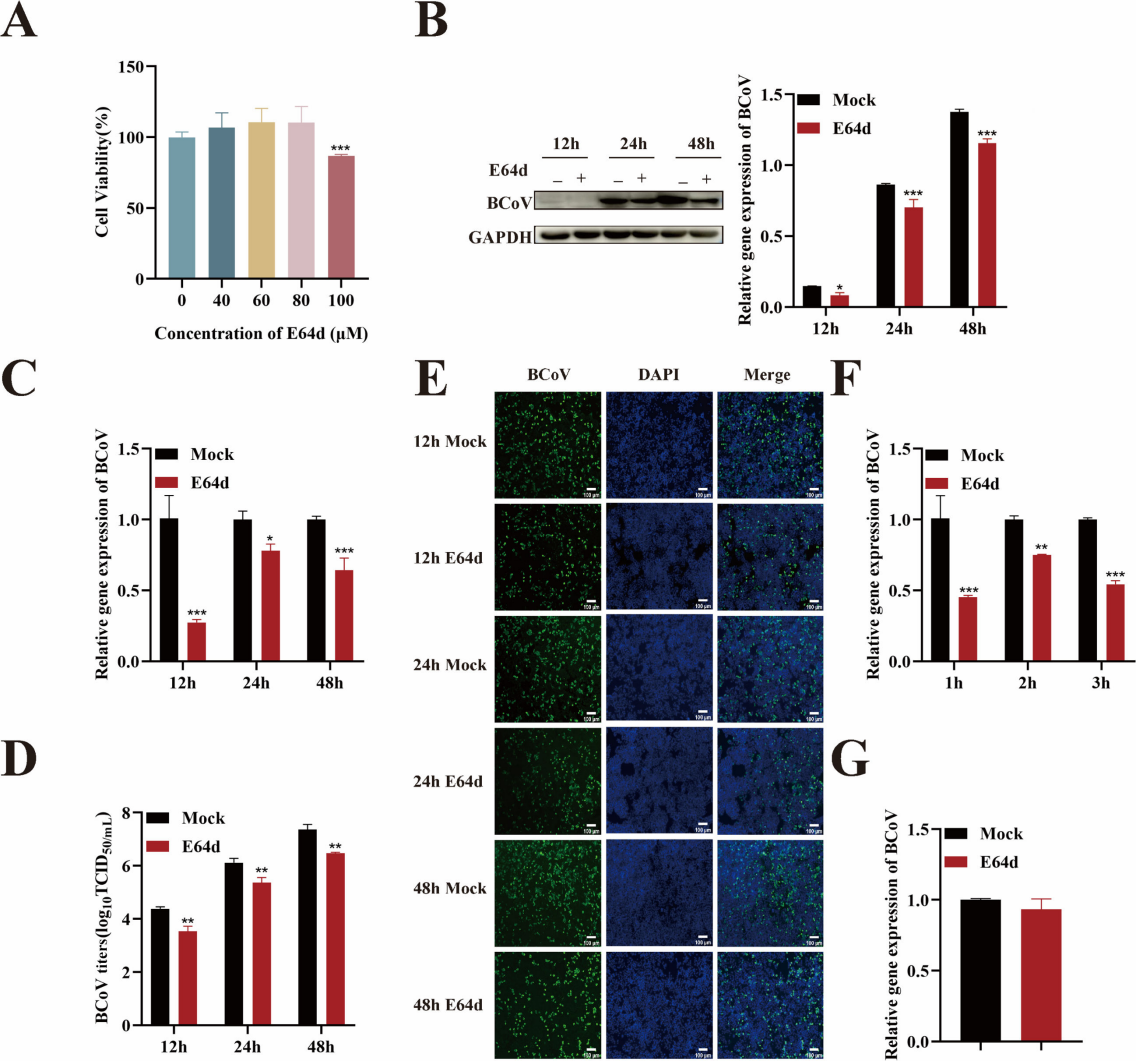
BCoV entry into HRT-18 cells depends on cathepsin. (**A**) The maximum safe concentrations of E64d were determined using the CCK-8 assay. (**B**) Western blot analysis was used to evaluate the BCoV N protein expression levels, with grayscale analysis performed and presented as a bar graph. (**C**) RT-qPCR was performed to assess the BCoV gene copy numbers. (**D**) TCID_50_ assay was used to measure the BCoV viral titers in the cell supernatant. (**E**) IFA was used to detect the number of BCoV-infected cells. Scale bar = 100 µm. (**F**) RT-qPCR was used to evaluate the effect of E64d on the viral entry. (**G**) RT-qPCR was used to evaluate the effect of E64d on BCoV attachment. Data are presented as the mean ± SD of three independent experiments (not significant, *P* > 0.05; *P < 0.05; **P < 0.01; ***P < 0.001).

To investigate the role of host cell cathepsins in BCoV entry, HRT-18 cells were treated with E64d, a broad-spectrum cathepsin inhibitor. E64d inhibits host cell cathepsins and thereby prevents the cleavage of the coronavirus S protein, which interferes with membrane fusion between the virus and the endosomal membrane, ultimately blocking viral entry. Firstly, CCK-8 assays determined that the maximum non-cytotoxic concentration of E64d in HRT-18 cells was 80 µM (Fig. 9A). Based on this result, HRT-18 cells were treated with 80 µM E64d for 24 h and subsequently infected with BCoV (MOI = 1) for 12, 24, and 48 h. Compared to untreated controls, E64d-treated cells showed significantly decreased viral RNA copy numbers, N protein expression, infection rates, and progeny virus titers as determined by RT-qPCR, Western blotting, IFA, and TCID_50_ (*P* < 0.05; Fig. 9B through E), suggesting that E64d effectively suppressed BCoV infection in HRT-18 cells. Then, cells were pretreated with E64d (80 µM) for 24 h and infected with BCoV (MOI = 5). Cell samples were collected during the attachment phase and at 1, 2, and 3 h post-entry. RT-qPCR analysis revealed that viral RNA copy numbers in the E64d-treated group were significantly reduced at all post-entry time points compared with the untreated group (*P* < 0.01; Fig. 9F), while no significant difference was observed during the attachment phase (*P* > 0.05; Fig. 9G). Together, the results demonstrated that cathepsin activity was essential for BCoV entry but dispensable for viral attachment.

### BCoV entry into HRT-18 cells is independent of TMPRSS2 activity

**Fig 10:**
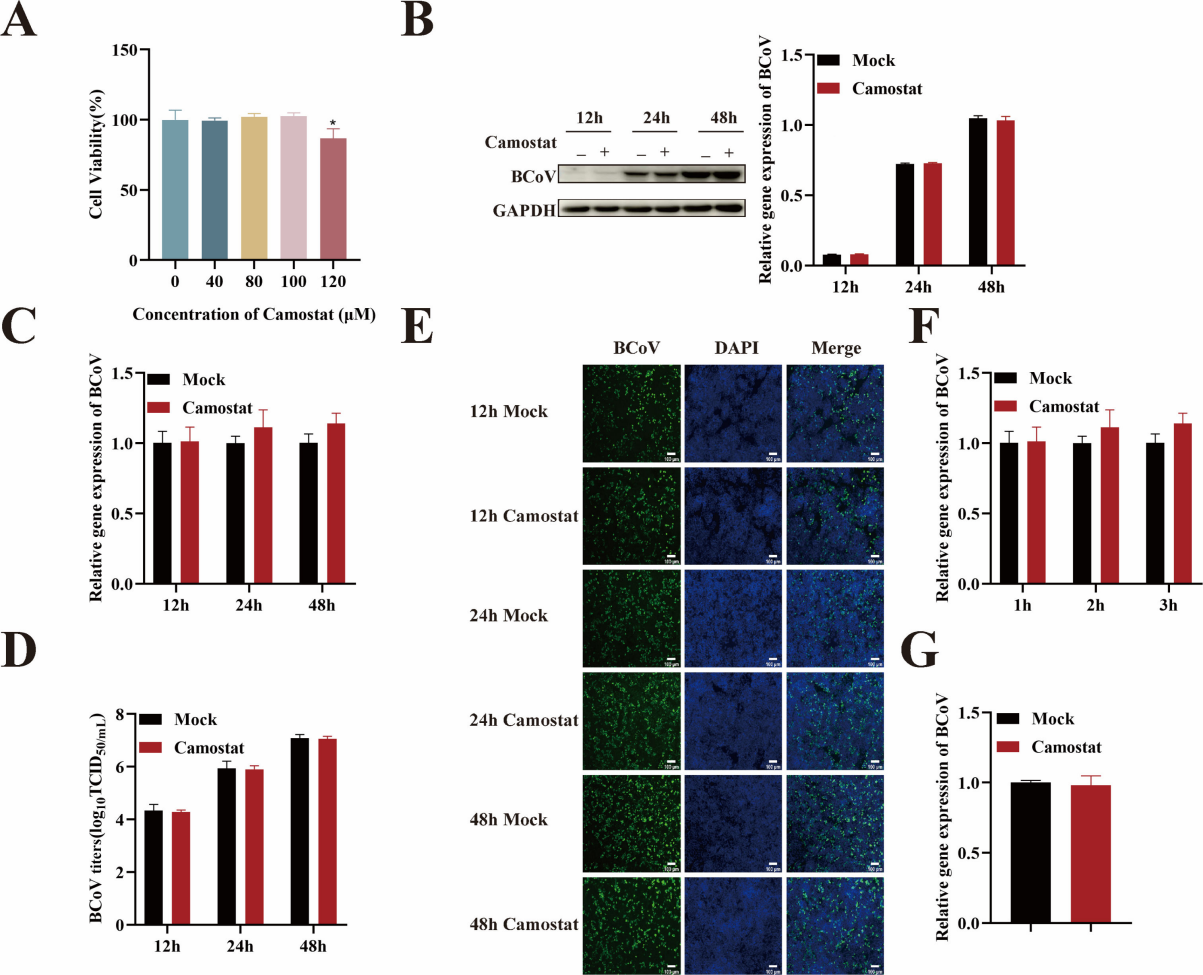
BCoV entry into HRT-18 cells is independent of TMPRSS2 activity. (**A**) The maximum safe concentrations of camostat were determined using the CCK-8 assay. (**B**) Western blot analysis was used to evaluate the BCoV N protein expression levels, with grayscale analysis performed and presented as a bar graph. (**C**) RT-qPCR was performed to assess the BCoV gene copy numbers. (**D**) TCID_50_ assay was used to measure the BCoV viral titers in the cell supernatant. (**E**) IFA was used to detect the number of BCoV-infected cells. (**F, G**) RT-qPCR was used to evaluate the effect of camostat on the viral entry and attachment. Scale bar = 100 µm. Data are presented as the mean ± SD of three independent experiments (not significant, *P* > 0.05).

To further investigate whether BCoV could utilize serine protease-mediated activation at the plasma membrane to enter HRT-18 cells, we focused on TMPRSS2, a host protease known to cleave the coronavirus S protein at the cell surface. Firstly, CCK-8 assays confirmed that camostat, a TMPRSS2 inhibitor, was non-cytotoxic to HRT-18 cells at concentrations up to 100 µM (Fig. 10A). HRT-18 cells were then pretreated with 100 µM camostat for 24 h prior to infection with BCoV (MOI = 1). Cells were collected at 12, 24, and 48 h post-infection and analyzed by RT-qPCR, Western blotting, IFA, and TCID_50_. Compared to the untreated group, camostat treatment did not result in significant differences in viral RNA copy numbers, N protein expression levels, or infectious titers at any of the examined time points (*P* > 0.05; Fig. 10B through E), indicating that TMPRSS2 inhibition does not affect BCoV infection. To further assess the role of TMPRSS2, we examined the effects of camostat on BCoV attachment and entry and found that camostat had no impact on either process (*P* > 0.05; Fig. 10F and G). These results suggest that BCoV entry into HRT-18 cells does not depend on TMPRSS2 activity.

### Live-cell imaging reveals that CPZ and SSAA09E3 block BCoV entry

**Fig 11:**
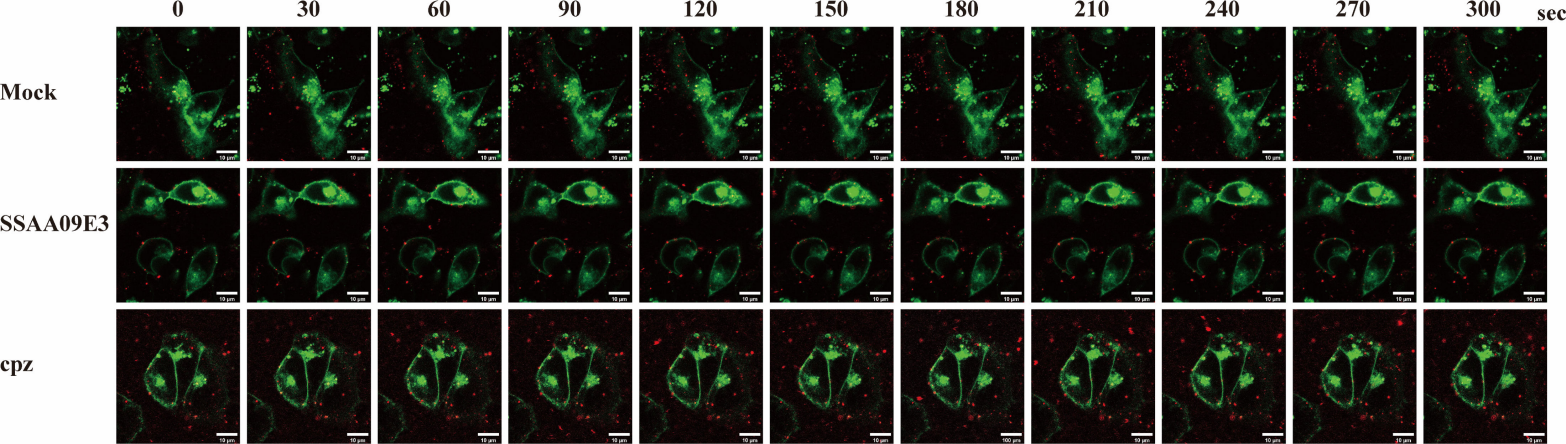
Visualization of BCoV entry into HRT-18 cells using DiD-labeled virus and DiO membrane staining. Scale bar = 10 µm.

Based on our previous findings that BCoV entry is dependent on CME and membrane fusion, we sought to further validate these pathways by directly visualizing viral entry. To this end, three experimental groups were established: a CPZ-treated group to inhibit CME, an SSAA09E3-treated group to block membrane fusion, and an untreated control group. HRT-18 cells were pretreated with CPZ or SSAA09E3 for 24 h, followed by labeling of the cell membrane with 3,3´-dioctadecyloxacarbocyanine perchlorate (DiO). The virus was labeled with the lipophilic dye 1,1′-dioctadecyl-3,3,3′,3′-tetramethylindodicarbocyanine (DiD). DiD-labeled BCoV was subsequently added and incubated with the cells at 4°C for 1 h. After incubation, the viral entry process was monitored for 5 min using a high-speed super-resolution confocal laser scanning microscope. In the untreated control group, most DiD-labeled BCoV particles gradually traversed the cell membrane and entered the cytoplasm (Fig. 11). In contrast, in both the CPZ- and SSAA09E3-treated groups, the majority of viral particles remained associated with the cell surface and failed to enter the cytoplasm (Fig. 11). These findings provide strong evidence that CPZ and SSAA09E3 effectively inhibited BCoV entry, thereby confirming the involvement of CME and membrane fusion in the entry process.

### The role of Rab proteins in BCoV infection of HRT-18 cells

**Fig 12:**
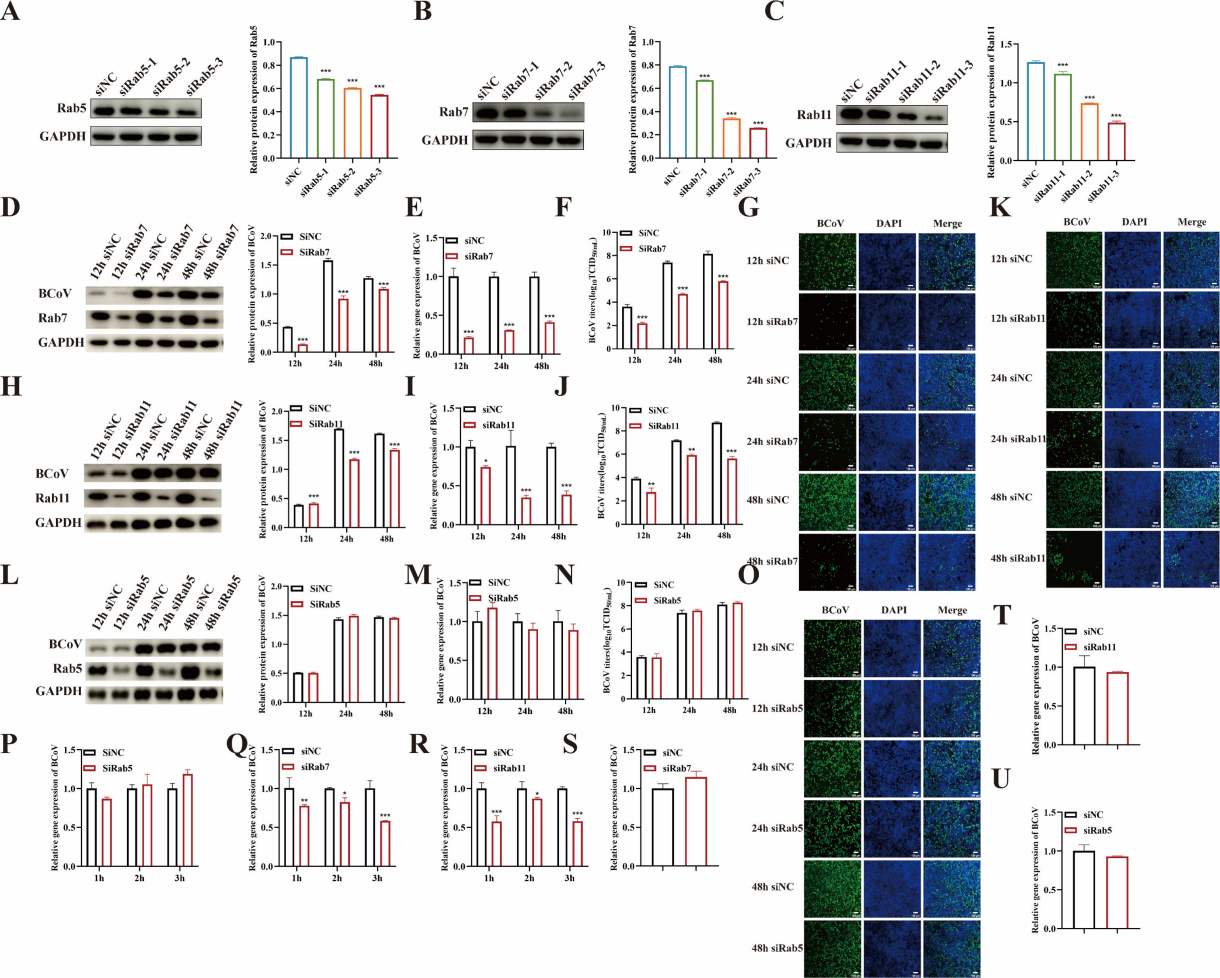
Rab7 and Rab11 are essential for BCoV endocytosis. (**A–C**) The siRNA silencing efficiency of Rab5, Rab7, and Rab11 was screened. (**D, E, F, G**) Silencing of Rab7 inhibits BCoV infection. HRT-18 cells were transfected with siRNA targeting Rab7 for 48 h, followed by BCoV infection for 12 h, 24 h, and 48 h. The effects of Rab7-silenced cells on BCoV infection were assessed by (**D**) Western blot, (**E**) RT-qPCR, (**F**) TCID_50_, and (**G**) IFA. (**H, I, J, K**) Rab11 silencing inhibits BCoV infection. HRT-18 cells were transfected with siRNA targeting Rab11 for 48 h, followed by BCoV infection for 12 h, 24 h, and 48 h. The effects of Rab11 silencing on BCoV infection were assessed by (**H**) Western blot, (**I**) RT-qPCR, (**J**) TCID_50_, and (**K**) IFA. (**L, M, N, O**) Silencing of Rab5 does not affect BCoV infection. HRT-18 cells were transfected with siRNA targeting Rab5 for 48 h, followed by BCoV infection for 12 h, 24 h, and 48 h. The effects of Rab5-silenced cells on BCoV infection were assessed by (**L**) Western blot, (**M**) RT-qPCR, (**N**) TCID_50_, and (**O**) IFA. (**P**) Rab5 silencing does not affect BCoV entry. After silencing of Rab5, cells were infected with BCoV for 1 h, 2 h, and 3 h, and BCoV gene copy number was measured by RT-qPCR. (**Q, R**) Rab7 and Rab11 silencing inhibits BCoV entry. After silencing of Rab7 and Rab11, cells were infected with BCoV for 1 h, 2 h, and 3 h, and BCoV gene copy number was measured by RT-qPCR. (**S, T, U**) RT-qPCR was used to evaluate the effects of Rab7-, Rab11-, and Rab5-targeting siRNAs on BCoV attachment. Data are presented as the mean ± SD of three independent experiments (not significant, *P* > 0.05; *P < 0.05; **P < 0.01; ***P < 0.001).

**Fig 13:**
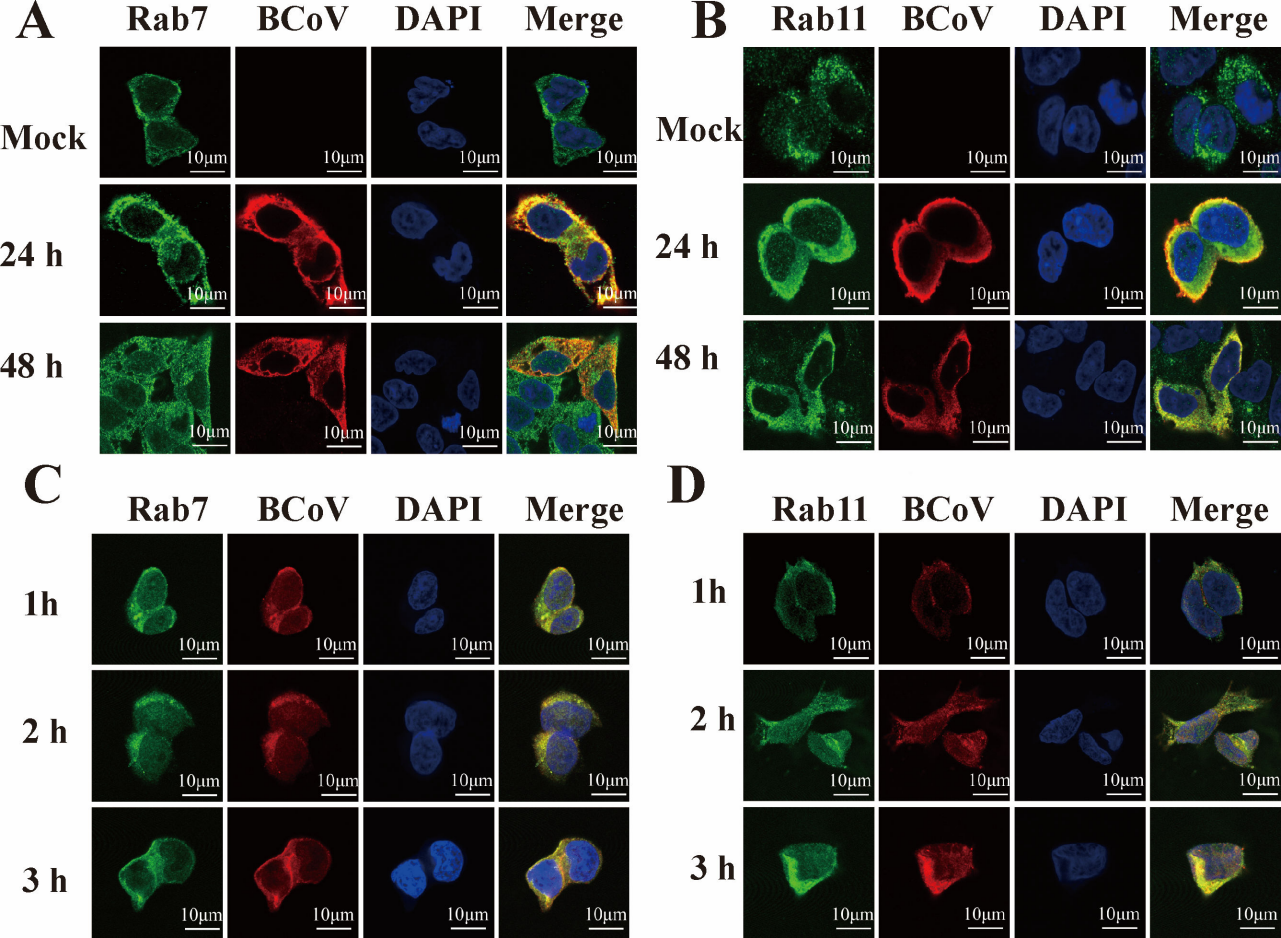
Colocalization of Rab7 and Rab11 with BCoV during the stage of infection and entry. (**A, B**) HRT-18 cells were infected with BCoV at an MOI of 1 and cultured for 12 h and 24 h, respectively, stained with rabbit anti-BCoV N antibody, mouse anti-Rab7 antibody (**A**), or mouse anti-Rab11 antibody (**B**), and examined by confocal microscopy. Scale bars, 10 µm. (**C, D**) HRT-18 cells were infected with BCoV at an MOI of 5 at 1 h, 2 h, and 3 h, respectively. (**C**) Cells were co-stained with anti-N and anti-Rab7 antibodies. (**D**) Cells were co-stained with anti-N and anti-Rab11 antibodies. Scale bars, 10 µm.

Small GTPase Rab proteins play critical roles in viral entry by regulating endosomal trafficking. Small GTPases, such as Rab5, Rab7, and Rab11, are key regulators of early endosomes, late endosomes, and recycling endosomes, respectively (16). Here, interference with the expression of Rab5, Rab7, and Rab11 was used to verify the role of Rab-mediated endosomal transport in BCoV entry. Firstly, the silencing efficiency was determined by Western blotting (Fig. 12A through C). Cells were transfected with siRab5, siRab7, siRab11, or siNC for 48 h, followed by BCoV infection (MOI = 1). Viral proliferation was comprehensively evaluated at 12, 24, and 48 h post-infection using RT-qPCR, Western blotting, IFA, and TCID_50_. The results showed that the BCoV gene copy numbers, protein expression levels, viral infection numbers, and progeny viral titers were significantly decreased in Rab7- (*P* < 0.001; Fig. 12D through G) and Rab11-silenced cells (*P* < 0.05; Fig. 12H through K), but not in Rab5-silenced cells (*P* > 0.05; Fig. 12L through O). To further analyze BCoV entry, the RT-qPCR analysis revealed that Rab7 and Rab11 silencing significantly reduced viral RNA copy numbers (*P* < 0.05; Fig. 12Q and R), whereas Rab5 silencing had no effect (*P* > 0.05; Fig. 12P). However, silencing Rab7, Rab11, and Rab5 had no effect on BCoV attachment (*P* > 0.05; Fig. 12S through U). Confocal microscopy further demonstrated co-localization of BCoV with Rab7 and Rab11 during entry (1, 2, and 3 h) and infection (24, 48 h) (Fig. 13A through D). Collectively, these findings demonstrate that BCoV trafficking via endosomes is dependent on Rab7 and Rab11.

## DISCUSSION

As obligate intracellular parasites, viral infectivity hinges on breaching host defense barriers and establishing efficient entry pathways (27). Elucidating the mechanisms of viral entry into cells is not only crucial for understanding the pathogenic mechanisms of viral infections but also contributes to the development of antiviral drugs. However, the way of BCoV entry into cells has not been reported. In this study, we provide the first evidence that BCoV enters HRT-18 cells via the membrane fusion and CME, requiring an acidic environment, dynamin, cholesterol, microtubules, cathepsins, and Rab7- and Rab11-mediated endosomal trafficking.

Currently, viral entry mechanisms are primarily categorized into two classical pathways: membrane fusion and receptor-mediated endocytosis (10). Coronaviruses, including SARS-CoV-2 (12), PEDV (15), and mouse hepatitis virus type 2 (28) enter cells via CME and membrane fusion. Based on this precedent, we hypothesized that BCoV enters HRT-18 cells via endocytosis and membrane fusion. Following endocytosis, an acidic environment is required for fusion between the viral envelope and endosomal membrane for most enveloped viruses (29). Here, two chemical inhibitors of endosomal acidification, NH_4_Cl and CQ, were employed to modulate the pH of endosomes. Data showed that both inhibitors markedly suppressed BCoV proliferation and entry, suggesting that an acidic environment is critical for BCoV entry into HRT-18 cells. To further investigate whether BCoV enters HRT-18 cells via CME and membrane fusion, cells were treated with SSAA09E3 (a membrane fusion inhibitor), CPZ (a CME inhibitor), and CLTC silencing by siRNA. The results demonstrated that treatment with SSAA09E3, CPZ, and CLTC silencing significantly inhibited BCoV proliferation and entry, suggesting that BCoV utilizes both membrane fusion and CME for entry. Membrane fusion is an essential step for the entry of enveloped viruses into host cells. During this process, viral particles recognize specific host cell receptors and enter susceptible cells through the fusion of the viral envelope with the host cell membrane (30). CME is a classical endocytic pathway and a major route for viral entry into cells. In this process, upon binding to host cell receptors, the virus triggers the recruitment of clathrin complexes to the inner side of the plasma membrane. This recruitment leads to the formation of invaginated structures that encapsulate viral particles, facilitating their internalization (31). To direct visualization of the effect of CPZ and SSAA09E3 on BCoV entry, live-cell imaging was performed and showed that both treatments inhibited the entry of DiD-labeled BCoV particles, which remained on the cell surface, further confirming that both CME and membrane fusion are essential for BCoV entry. Dynamin is a GTPase that assembles into a ring at the neck of the invaginated pit, and through its GTPase activity, it pinches off the endocytic vesicle from the cell membrane to form a clathrin- and caveolae-coated vesicle (32). Studies have shown that dynamin is required for the entry of multiple viruses, such as African swine fever virus (33), herpes simplex virus type 1 (HSV-1) (34), and rabies virus (35). Given the role of CME in BCoV entry, the role of dynamin in this process was further investigated. In this study, the use of a dynamin inhibitor and dynamin-targeting siRNA revealed that dynamin plays an important role in BCoV invasion of HRT-18 cells.

Emerging evidence has revealed that multiple viruses, including SARS-CoV-2 (15, 36), human immunodeficiency virus (HIV) (37), Seneca Valley virus (SVV) (38), influenza virus (39), and Newcastle disease virus (40), enter cells via more than one endocytic pathway. It has also been found that such viruses invade different susceptible cells in different ways. For example, PDCoV enters PK-15 cells through the CavME (13), while in IPI-2I cells, it exploits both macropinocytosis and CME (14). Classical swine fever virus invades the porcine alveolar macrophage cell line 3D4/21 via CavME (41), but invades PK-15 cells via CME (42). To investigate whether CavME and micropinocytosis contribute to BCoV entry into HRT-18 cells, we treated cells with nystatin (a CavME inhibitor) and blebbistatin (a macropinocytosis inhibitor) and showed these two inhibitors did not block virus entry, suggesting that BCoV entry into HRT-18 cells was dependent on CME rather than CavME and micropinocytosis.

Cholesterol plays a crucial role in the entry of enveloped and non-enveloped viruses through diverse mechanisms (43). For influenza virus (44) and dengue virus (45), cholesterol is involved in the formation of lipid rafts in the cell membrane, which facilitates precise docking of the virus with cell surface receptors. For hepatitis C virus (46) and lymphocytic choriomeningitis virus (47), cholesterol influences membrane fluidity and rigidity, promoting membrane invagination and the formation of endocytic vesicles that encapsulate the virus during entry. Additionally, cholesterol regulates the fluidity and permeability of both the cell membrane and the viral envelope, enhancing membrane fusion. This mechanism is observed in viruses such as HIV (37) and SARS-CoV-2 (36) during their entry into host cells. Given the critical role of cholesterol in the entry of many viruses, it is reasonable to investigate whether cholesterol similarly contributes to BCoV entry. Here, the cholesterol-depleting agent MβCD was used to assess its potential impact on BCoV entry into HRT-18 cells. The results revealed that depletion of cell membrane cholesterol significantly blocked BCoV entry.

The microtubule plays indispensable roles in viral entry through two distinct mechanisms. First, viruses utilize microtubules to facilitate binding to host cell surface receptors (48). Multiple viruses, including vaccinia virus (49), HSV-1 (50), West Nile virus (WNV) (51), and respiratory syncytial virus (52), hijack microtubules for directional trafficking during entry. Our investigation demonstrated that pretreatment with the microtubule-disrupting agent colchicine significantly inhibited BCoV entry into HRT-18 cells, suggesting that microtubules are essential for BCoV entry.

Cathepsins are lysosomal cysteine proteases that facilitate the entry of various enveloped viruses by cleaving viral glycoproteins within endosomes. This proteolytic activation is essential for triggering membrane fusion between the viral envelope and the host endosomal membrane, enabling the release of the viral genome into the cytoplasm (53). Several viruses, including Ebola virus (54), SARS-CoV (55), and MERS-CoV (56), rely on cathepsin-mediated cleavage of their surface glycoproteins to mediate endosomal fusion. In our study, treatment of HRT-18 cells with E64d, a broad-spectrum cathepsins inhibitor, significantly inhibited BCoV entry. These findings indicate that cathepsin activity is essential for BCoV entry, likely by enabling spike protein activation and membrane fusion within endosomes.

TMPRSS2 facilitates viral entry by cleaving and activating S proteins at the plasma membrane, thereby promoting direct fusion between the viral envelope and the host cell membrane. However, in this study, inhibition of TMPRSS2 activity with camostat did not suppress viral entry or infection, indicating that TMPRSS2 was not required for BCoV-mediated membrane fusion. This entry mechanism is consistent with reports on other coronaviruses that do not efficiently exploit TMPRSS2 and instead rely on endosomal proteases for S protein activation (57, 58).

Endosomal trafficking represents a critical pathway for viral internalization following entry, encompassing sequential progression through early endosomes, late endosomes, and recycling endosomes (16). These endosomes are mediated by Rab GTPases, with Rab5, Rab7, and Rab11 specifically orchestrating trafficking in early endosomes, late endosomes, and recycling endosomes (16). Coronaviruses, including porcine enteric alphacoronavirus (59) and infectious bronchitis virus (60), exemplify this paradigm by entering cells via clathrin-mediated endocytosis and undergoing sequential maturation through early and late endosomes. Here, silencing of Rab5, Rab7, and Rab11 by siRNA was performed to investigate the effect of endosomal trafficking on BCoV entry. The results showed that silencing of Rab7 and Rab11 potently inhibited BCoV entry into HRT-18 cells, while siRab5 had no significant effect on BCoV entry. Laser confocal microscopy further revealed the colocalization of BCoV with Rab7 and Rab11 during viral entry steps, confirming that BCoV traffics through late and recycling endosomes. Previous studies reported that porcine sapelovirus entry requires Rab7-dependent late endosomes and Rab11-dependent recycling endosomes (61), which is consistent with our findings. Notably, emerging studies revealed that SVV (62) and bluetongue virus (63) exclusively hijack late endosomes (Rab7) during entry, bypassing early endosomes.

In summary, this study revealed that BCoV enters HRT-18 cells via membrane fusion and CME; after endocytosis, the virion is transported from late and recycling endosomes but not early endosomes. Dynamin, cholesterol, microtubules, cathepsins, and an acidic environment are also required for BCoV entry.

## MATERIALS AND METHODS

### Cells and virus

HRT-18 cells were obtained from the American Type Culture Collection and cultured in Dulbecco’s Modified Eagle’s Medium (DMEM) (Gibco, Grand Island, NY, USA) supplemented with 10% fetal bovine serum (FBS) (Gibco, Grand Island, NY, USA) and 1% penicillin-streptomycin (Sigma-Aldrich, St. Louis, MO, USA). The cells were maintained in a 37°C incubator with 5% CO_2_. Bovine coronavirus (GenBank: OP866728.1) was isolated and preserved in our laboratory.

### Inhibitors

The inhibitors used in this study included SSAA09E3 (Cat HY-138102, MedChemExpress), a novel inhibitor that blocks the fusion of the viral membrane with the host cell membrane; CPZ (Cat C0982, Sigma), a clathrin-mediated endocytosis inhibitor; nystatin (Cat 475914, Sigma), a caveolae inhibitor that acts as a sterol-binding agent disrupting caveolae; blebbistatin (Cat 203391, Sigma), an inhibitor of micropinocytosis; dynasore (Cat T1848, TargetMol), a dynamin inhibitor; MβCD (Cat T4072, TargetMol), a cholesterol depletion inhibitor; chloroquine (Cat S6999, Selleck) and NH_4_Cl (Cat A9434, Sigma), a potent inhibitor of V-ATPase and a specific inhibitor of acidification of endosomal vesicles; colchicine (Cat HY-16569, MedChemExpress), which inhibits the polymerization of tubulin; E64d (Cat S7393, Selleck), a cathepsin inhibitor; and camostat (Cat HY-13512, MedChemExpress), a TMPRSS2 inhibitor.

### Cell viability assay

Cell viability was assessed using the CCK-8 assay. HRT-18 cells were seeded into 96-well plates and inoculated with SSAA09E3 (5, 10, 20, 40 µM), CPZ (5, 10, 20, 40 µM), nystatin (10, 20, 40, 80 µM), blebbistatin (5, 10, 20, 40 µM), dynasore (5, 10, 20, 40 µM), NH_4_Cl (10, 20, 40, 80 mM), chloroquine (20, 40, 80, 100 µM), MβCD (0.63, 1.25, 2.5, 5 mg/mL), colchicine (200, 400, 600, 800 nM), E64d (40, 60, 80, 100 µM), or camostat (40, 80, 100, 120 µM) for 48 h at 37°C. Then, 10 µL of CCK-8 reagent was added to each well for 1 h at 37°C. The absorbance values were recorded at 450 nm using an Infinite M200 Pro system (Tecan, Männedorf, Switzerland). A viability of over 90% without morphological changes was considered the safe concentration.

### Western blotting

Proteins were extracted from HRT-18 cells using RIPA lysis buffer (Cat P0013B, Beyotime) and quantified using a BCA Protein Quantification Kit (Cat P0012, Beyotime). Protein samples were separated by SDS-PAGE and transferred onto a 0.22 µm PVDF membrane (Cat ISEQ00010, Merck Millipore). The membrane was blocked at room temperature for 2 h in TBST containing 5% non-fat milk. The primary antibodies used were GAPDH (Cat YM3029, Immunoway), Rab5 (Cat 66339-1-Ig, Proteintech), Rab7 (Cat R8779-25UL, Sigma-Aldrich), and Rab11 (Cat 67902-1-Ig, Proteintech). The anti-N protein antibody of BCoV was prepared and stored in our laboratory. The primary antibody was incubated overnight at 4°C. The HRP-conjugated goat anti-mouse/rabbit IgG (H+L) secondary antibody (Cat RS0001/RS0002, Immunoway) was incubated at room temperature for 1 h at a dilution of 1:12,000. After washing the membrane with TBST, ECL detection was performed. Protein bands were detected and analyzed using the Amersham ImageQuant 800 Western blot imaging system (Cytiva, Sweden), and grayscale analysis was performed using ImageJ.

### TCID_50_

The cell supernatants were serially diluted and used to infect HRT-18 cells in 96-well plates. The cells were incubated at 37°C in a 5% CO_2_ incubator for 48 h. The cells were fixed with 4% paraformaldehyde for 20 min, washed with PBS, and then permeabilized with 0.3% Triton X-100 for 15 min. Afterward, the cells were blocked with 5% skim milk for 3 h. The BCoV N protein antibody (1:100) was incubated overnight at 4°C, and the goat anti-rabbit IgG (H+L) (Alexa Fluor 488) (Cat RS3211, Immunoway) antibody (1:200) was incubated for 1 h at room temperature in the dark. Nuclei were stained with Hoechst 33342 for 10 min. The fluorescence-positive wells were observed under an inverted fluorescence microscope (Axio Observer, ZEISS, Germany). Finally, the TCID_50_ was calculated by the Reed-Muench method.

### Confocal microscopy

HRT-18 cells were seeded onto cell culture coverslips in 24-well plates and incubated for 12 h at 37°C in a 5% CO_2_ incubator. (i) Cells were infected with BCoV for 1, 2, and 3 h, and the co-localization of CLTC, Rab7, and Rab11 with BCoV was detected. (ii) Cells were infected with BCoV for 24 and 48 h, and the co-localization of Rab7 and Rab11 with BCoV was detected. (iii) HRT-18 cells were treated with colchicine for 24 h to verify its inhibition of microtubule polymerization.

Cells were then washed three times with PBS and fixed with 4% paraformaldehyde at room temperature. Cells were then permeabilized with 0.3% Triton X-100 and blocked with 5% skim milk. The cells were incubated with BCoV antibody and mouse anti-Rab7 (Cat R8779-25UL, Sigma-Aldrich), mouse anti-Rab11 (Cat 67902-1-Ig, Proteintech), mouse anti-tubulin (Cat 66240-1-Ig, Proteintech), or mouse anti-CLTC (Cat 66339-1-Ig, Proteintech) overnight at 4°C. After three washes with PBS, the secondary antibodies Alexa Fluor 488 Anti-Mouse and Alexa Fluor 594 Anti-Rabbit were incubated for 1 h at room temperature in the dark. Subsequently, cells were incubated with DAPI (Cat BL105A, Biosharp) at 37°C for 10 min and washed three times with PBS. Finally, images were captured using a Leica TCS SP8 laser scanning confocal microscope (LSM510 META, ZEISS, Germany).

### Real-time quantitative PCR

Total RNA was extracted using the TRIzol method, and the RNA concentration was measured using a NanoDrop One (Thermo Fisher Scientific, Waltham, MA, USA). The RNA was reverse transcribed into cDNA using the Evo M-MLV Reverse Transcription Kit (Cat AG11728, Accurate Biology) according to the manufacturer’s instructions. GAPDH was used as the internal reference gene, and the expression changes of BCoV, Rab5, Rab7, Rab11, dynamin, and CLTC genes were analyzed. Primer sequences are shown in Table 1. Real-time quantitative PCR was performed using the SYBR Green Pro Taq HS Premix qPCR Kit (Cat AG11701, Accurate Biology). The reaction mixture had a final volume of 20 µL, with the following amplification program: 95°C for 30 seconds, followed by 40 cycles of 95°C for 5 seconds and 60°C for 30 seconds. The melt curve analysis was conducted using the CFX Connect Real-Time PCR Detection System (Bio-Rad, Hercules, CA, USA). Data were analyzed using the 2^–ΔΔCT^ method.

**TABLE 1:** Primers used for RT-qPCR

Primer	Sequence (5´–3´)
GAPDH-F	AAGGCTGTGGGCAAGG
GAPDH-R	TGGAGGAGTGGGTGTCG
BCoV-F	CGTTCTGGTAATGGCATCCTTA
BCoV-R	GTTTGCTTGGGTTGAGCTCTTCTA
Rab5-F	CCGACCTAGCAAATAAAAGAGC
Rab5-R	AGCGGATGTCTCCATGAATAAT
Rab7-F	GCATCCTAGCTTTTGATGTCAC
Rab7-R	CATTCTTGGCACTGACTTCAAA
Rab11-F	TTGTGGGCAATAAGAGTGATCT
Rab11-R	TGGAACATGAATAGGAACCACA
CLTC-F	CAATGGACCAAATAATGC
CLTC-R	GTGAACCAGGGTAGATGC
Dynamin-F	CAAGGATGAGGAGGAGAAAG
Dynamin-R	GTGTTGAAGATGGCGAAGA

### siRNA and transfection

The siRNA was synthesized by Qingke Bio (Beijing, China), with three siRNA sequences designed for each target gene (dynamin, CLTC, Rab5, Rab7, and Rab11). The sequences are shown in Table 2. The siNC sequence was supplied and synthesized by the company (Qingke Bio, Beijing, China). HRT-18 cells were seeded into six-well plates, and transfection was performed when the cell confluence reached 60%. A total of 1.6 mL of DMEM medium containing 10% FBS was added to each well. Two sterile, clean centrifuge tubes were prepared. Then, 125 µL of Opti-MEM medium was added to each tube, and 100 pmol of siRNA was added to one tube, which was mixed gently using a pipette. To the second tube, 5 µL of Lipo6000 (Cat C0526FT, Beyotime) transfection reagent was added and mixed gently. After incubation at room temperature for 5 min, the siRNA-containing solution was gently added to the Lipo6000 reagent-containing solution and mixed gently. The mixture was then incubated at room temperature for 15 min. The cells were incubated at 37°C for 48 h, and the interference efficiency was verified by Western blot. The siRNA with the highest interference efficiency was used for subsequent experiments.

**TABLE 2:** Sequences of siRNAs used in this study

Target gene	Sense	Antisense
siRab5A-1	CAGCUGGUCAAGAACGAUA	UAUCGUUCUUGACCAGCUG
siRab5A-2	GCAAGUCCUAACAUUGUAA	UUACAAUGUUAGGACUUGC
siRab5A-3	GAACUUCAGAGGCAAGCAA	UUGCUUGCCUCUGAAGUUC
siRab7-1	GGUGUAGAGAGAAAGAUAU	AUAUCUUUCUCUCUACACC
siRab7-2	UAUCGUUCUUGACCAGCUG	CCAUGGUGUCCACGUUCUA
siRab7-3	GCUGCGUUCUGGUAUUUGA	UCAAAUACCAGAACGCAGC
siRab11-1	CAAGAGCGAUAUCGAGCUA	UAGCUCGAUAUCGCUCUUG
siRab11-2	GUGUUGGAAAGAGUAAUCU	AGAUUACUCUUUCCAACAC
siRab11-3	GCAACAAUGUGGUUCCUAU	AUAGGAACCACAUUGUUGC
siCLTC-1	CAGAUGCUAUUCUAGGCAA	UUGCCUAGAAUAGCAUCUG
siCLTC-2	GGCCUGUGUUGCUUAUGAA	UUCAUAAGCAACACAGGCC
siCLTC-3	ACGAAUUGGCAGAAUCUGGGC	CCAGAUUCUGCCAAUUCGUUU
siDynamin-1	CAUUCGAGGCCAUUGUGAA	UUCACAAUGGCCUCGAAUG
siDynamin-2	GAGCUAAUCAAUACAGUUA	UAACUGUAUUGAUUAGCUC
siDynamin-3	GAACGAAGGACCAGAUUCU	AGAAUCUGGUCCUUCGUUC

### Virus invasion assay

For the virus attachment assay, cells were pretreated for 24 h with the maximum non-cytotoxic concentrations of chemical inhibitors (SSAA09E3, chlorpromazine, nystatin, blebbistatin, dynasore, NH_4_Cl, MβCD, chloroquine, colchicine, E64d, and camostat). Alternatively, cells were transfected with siRNAs targeting clathrin, dynamin, Rab5, Rab7, or Rab11. Following pretreatment, the cells were incubated with BCoV at an MOI of 5 at 4°C for 1 h to permit viral attachment without internalization. Unbound virus was removed by washing the cells three times with ice-cold PBS. Total RNA was then extracted, and viral genomic RNA copy numbers were quantified by RT-qPCR to assess the level of virus attachment.

For the viral entry assay, cells were pretreated for 24 h with the maximum non-cytotoxic concentrations of the respective compounds or transfected with siRNAs. Following pretreatment, the cells were infected with BCoV at a MOI of 5 and incubated at 4°C for 1 h to allow viral attachment without internalization. Unbound virus was removed by washing the cells three times with ice-cold PBS, after which the cells were incubated at 37°C for 1, 2, or 3 h to permit viral internalization. Subsequently, the cells were washed again three times with ice-cold PBS, and total RNA was extracted. Viral genomic RNA copy numbers were subsequently quantified by RT-qPCR.

### Fluorescent labeling and imaging of BCoV entry into HRT-18 cells

The virus was labeled with the lipophilic dye DiD (Thermo Fisher, V22887, USA), which incorporates into viral envelopes. DiD was added to the viral suspension and incubated at room temperature for 120 min. Unincorporated dye was subsequently removed using NAP-10 desalting columns (Cytiva, GE Healthcare). The DiD-labeled BCoV was then filtered through a 0.22 µm membrane filter (Millipore) and stored at −80°C until use in viral tracking assays.

To enable direct visualization of BCoV entry, HRT-18 cell membranes were labeled with the lipophilic dye DiO (Thermo Fisher, V22886, USA). After removal of excess dye, DiD-labeled BCoV was added and incubated with the cells at 4°C for 1 h. The entry process was subsequently imaged using a high-speed, super-resolution confocal laser scanning microscope (LSM980 with Airyscan2, Carl Zeiss Microscopy GmbH, Germany) equipped with a 63× oil immersion objective (NA 1.4).

### Flow cytometry and fluorescence microscopy for quantitative and visual assessment of BCoV-induced syncytium formation

Cell membranes were labeled with the lipophilic dye DiD, which integrates into the lipid bilayer. Upon membrane fusion between adjacent cells, the labeled membranes merge, resulting in larger multinucleated cells (syncytia) that exhibit enhanced and aggregated DiD fluorescence signals. Flow cytometry detects these changes as an increase in mean fluorescence intensity (MFI), reflecting the extent of membrane fusion. This method enables high-throughput, quantitative analysis of fusion events at the single-cell level. The experiment included six groups: (i) uninfected and untreated, (ii) uninfected and treated with SSAA09E3, (iii) BCoV-infected for 3 h without SSAA09E3, (iv) BCoV-infected for 3 h with SSAA09E3, (v) BCoV-infected for 24 h without SSAA09E3, and (vi) BCoV-infected for 24 h with SSAA09E3. Following treatment, HRT-18 cells were labeled with DiD to stain the plasma membrane, washed to remove excess dye, digested with trypsin (without EDTA), and analyzed by flow cytometry (BD high-speed cell sorter, APC channel). Data were processed using FlowJo v.10.

To directly visualize syncytium formation, fluorescence microscopy was performed. HRT-18 cells were allocated into three experimental groups as follows: (i) uninfected control cells (mock group), (ii) BCoV-infected cells, and (iii) BCoV-infected cells with SSAA09E3 treatment. Syncytium formation was assessed by observing overall morphology with bright-field microscopy and by examining the BCoV N protein and nuclei with fluorescence microscopy.

### Data analysis

All experiments were performed at least three times, with consistent results across the replicates. Data were presented as mean ± SD. Statistical differences for all data were determined using one-way analysis of variance or Student’s *t*-test using GraphPad Prism 8.0 software. *P* < 0.05 was considered statistically significant, and *P* > 0.05 was deemed not significant.

## Data Availability

The authors confirm that all data supporting the findings of this study are available within the article.
